# The evolution of competition and policing: opposing selection within and among groups

**DOI:** 10.1186/1471-2148-7-203

**Published:** 2007-10-25

**Authors:** Yaniv Brandvain, Michael J Wade

**Affiliations:** 1 Department of Biology, Indiana University – Bloomington, IN, USA

## Abstract

**Background:**

Although selection favors exploitative competition within groups, a group of hypercompetitive individuals may be less productive than a cooperative group. When competition is costly for group fitness, among-group selection can favor groups with 'policing' individuals who reduce within-group competition at a cost to their own fitness, or groups of individuals who restrain their competitive intensity ('self policing'). We examine these possibilities in a series of explicit population-genetic models.

**Results:**

By comparing results from models of half and full sib structured populations, we find that increased relatedness increases the strength of among-group selection against competition genotypes, and increases the strength of among group selection favoring policing genotypes. However, the strength of selection favoring costly policing behavior also increases with increased levels of competition. When levels of competition and policing feedback on one another, groups with lower levels of relatedness can favor higher levels of costly policing.

**Conclusion:**

The result of the joint selection on policing and competition leads to results different from those based on the evolution of policing alone. Our model makes 'long term' predictions equivalent to those of optimization models, but we also show the existence of protected polymorphisms of police and civilians, as well as competitors and non-competitors.

## Background

A major impediment to the evolution of productive social interactions is what Hardin [1] termed "The Tragedy of the Commons" (TOC). Because individual self-interest favors unfettered exploitation of a resource, whenever two or more individuals share a common environment and make simultaneous demands on its resources, conditions are ripe for the TOC. Opportunities for the TOC include groups of microbes living in the same host, offspring within the same family, and genes in the same organism [2]. Wright ([3] p. 129), Wynne-Edwards ([4] p. 623) and Hamilton [5] viewed the TOC as the problem of controlling cheaters, lest they lead to the destruction of the group (see also [6-8]).

Resolution of this tension between the interests of the group and the individual allows major evolutionary transitions, wherein communities of individuals merge into a single functional unit [8-11]. The processes proposed for resolving this tension are kin selection for altruism and enforced cooperation via policing. These are viewed as alternatives, since the former is facilitated by high levels of genetic relatedness among group members, while the latter is favored by low levels of relatedness. Although group selection works best with high levels of relatedness, "contrary to simple expectation" ([12] p. 42), policing theory [13,14] and data from vespid wasps [15,16] indicate that the highest levels of policing occur in groups with the lowest levels of genetic relatedness. Hence, policing is viewed as a mechanism for integrating groups of unrelated individuals, while altruism is seen a mechanism for cohesion in related groups [2,12-14,17,18].

In this paper, we use explicit population genetic models to show that costly policing is favored by selection among groups and opposed by selection within groups in proportion to relatedness among group members in the same manner as kin-selected altruism: although altruistic and enforced cooperation may be alternative mechanisms for group cohesion, they evolve by the same evolutionary process. We examine the evolution of individual competitive ability and policing in the first two-trait model of individual and group selection. Individual selection within groups favors enhanced competitive ability but opposes costly policing. Conversely, group selection opposes the evolution of competitive ability but favors the evolution of policing.

Unlike previous optimization treatments of policing, our explicit genetic model reveals stable interior equilibria as well as the rate of progress toward them. Owing to the multiplicative nature of the group-mean fitness function (a pres [13]), many combinations of competition and policing have equal fitnesses. Although mutation analysis reveals that these interior equilibria are invasible and can move toward the global optima found by Frank [13], evolutionary progress toward the global equilibrium is exceedingly slow, on the order of the *square *of the inverse of the mutation rate, because it depends upon the order of occurrence of competition and policing mutations. These global optima may exist but few species will endure long enough to guarantee arrival at them.

Ratnieks [19] coined the term, 'policing' in the context of social insect colonies where worker females lay haploid, male eggs that are often eaten by other workers. Policing behaviors can mitigate the individually beneficial and the communally detrimental effects of individual selfishness ([2,13,14,20]). When individuals engage in mutual policing at some cost to themselves, the group as a whole functions as an evolutionary unit, as in the social insects, where "Worker policing ... may be selected due to the colony-level benefit of conflict suppression" ([21] p. 169). Additionally, policing is thought to be particularly important in the maintenance of sociality in higher primates. Alexander [22] argued that socially imposed rules and traditions favor group-level efficiency in humans, and Flack and colleagues have found evidence for the importance of policing in the coherence of groups of pigtailed macaques [20,23].

Frank [2,13,14,17,18] was the first to formally examine the co-evolutionary interplay between competitive and policing behaviors. In the simplest version of his model, Frank [13] envisions that each individual has two quantitative traits, policing and competition. The fitness, w_ij_, of the j^th ^individual in the i^th ^group is given by the function

wij=(ai.−caij+(zijzi.)(1−ai.))(1−zi.(1−ai.))
 MathType@MTEF@5@5@+=feaafiart1ev1aaatCvAUfKttLearuWrP9MDH5MBPbIqV92AaeXatLxBI9gBaebbnrfifHhDYfgasaacH8akY=wiFfYdH8Gipec8Eeeu0xXdbba9frFj0=OqFfea0dXdd9vqai=hGuQ8kuc9pgc9s8qqaq=dirpe0xb9q8qiLsFr0=vr0=vr0dc8meaabaqaciaacaGaaeqabaqabeGadaaakeaacqWG3bWDdaWgaaWcbaGaemyAaKMaemOAaOgabeaakiabg2da9maabmaabaGaemyyae2aaSbaaSqaaiabdMgaPjabc6caUaqabaGccqGHsislcqWGJbWycqWGHbqydaWgaaWcbaGaemyAaKMaemOAaOgabeaakiabgUcaRmaabmaabaWaaSaaaeaacqWG6bGEdaWgaaWcbaGaemyAaKMaemOAaOgabeaaaOqaaiabdQha6naaBaaaleaacqWGPbqAcqGGUaGlaeqaaaaaaOGaayjkaiaawMcaamaabmaabaGaeGymaeJaeyOeI0Iaemyyae2aaSbaaSqaaiabdMgaPjabc6caUaqabaaakiaawIcacaGLPaaaaiaawIcacaGLPaaadaqadaqaaiabigdaXiabgkHiTiabdQha6naaBaaaleaacqWGPbqAcqGGUaGlaeqaaOWaaeWaaeaacqaIXaqmcqGHsislcqWGHbqydaWgaaWcbaGaemyAaKMaeiOla4cabeaaaOGaayjkaiaawMcaaaGaayjkaiaawMcaaaaa@5E51@

where z_ij _is the competitive ability of the j^th ^individual and z_i. _is the mean competitive ability of individuals in group i. Similarly, a_ij _is the amount of policing by the j^th ^individual in the i^th ^group and a_i. _is the average level of policing within group i. The constant, c, represents the cost of policing to the individual and it is assumed to be the same for all individuals. Individual fitness increases as z_ij _increases and is favored by selection within groups. However, mean group fitness is a declining function of mean z_i _and is opposed by among group selection.

Analyzing the optima of eq. [1], Frank found that: (1) mean competitive ability decreases with increasing relatedness; and, (2) mean level of policing increases with increased competition. We use Frank's fitness model in our investigation of the evolution of competition and policing in populations composed of either full or half sib families. We examine these different levels of relatedness in order to explore directly how relatedness affects the evolution of policing and competition. We partition total selection into within and among group components [24] to make the levels of selection explicit and show how the major findings of Frank [13] emerge from the conflicting forces of selection within and between groups. Unlike Frank [11] we find many stable interior equilibria, in which extreme competitors coexist with less competitive genotypes, and effective police coexist with civilians; suggesting that a population need not necessarily arrive at the monomorphic state predicted by Frank [13]. Moreover, we find that like multiplicative selection functions of two-locus individual selection, joint selection on competitive and policing alleles does not generate linkage-disequilibrium, and thus the hyper-competitive genotypes are equally likely to be police or civilians.

## Methods and Results

### General Approach

In our model, policing and competition only occur within sibling groups. This can be visualized as competition over a resource proximal to the location where offspring are raised, or competition over maternal resources. After siblings mature, they leave this locale, mix with other members of the population and mate randomly, with no further selection on levels of competition or policing.

To further simplify analysis, we assume a large population in Hardy-Weinberg equilibrium, and we ignore the effects of mutation, migration, and random genetic drift. For each model, we identify all parental mating types and their respective frequencies, the offspring genotypes from each mating type, and the change in genotype frequencies within each family that result from the fitness costs and benefits of competition and policing. Next, we calculate mean absolute fitness of each family. From these values, we calculate the changes in gene frequency due to selection within sib groups, selection among sib groups, and the total change in gene frequency per generation. We complement our technical treatments by concluding each model with a qualitative summary of our findings.

### Model 1: The Evolution of Competitive Ability

We envision a population with no policing where an individual's level of competition is controlled by one locus with two alleles with additive affects. Let allele A occur in frequency p and the alternative allele, a, in frequency q = (1 - p), so that, after random mating, *AA*, *Aa*, and *aa *individuals are in frequencies p^2^, 2pq, and q^2^, respectively. Let *AA *individuals have a baseline competitive ability denoted by z_0_. Adding an *a *allele to the genotype, increases competitive ability by an amount, z_a_. Thus, Aa individuals compete at the level z_0 _+ z_a_, while aa individuals compete at the level, z_0 _+ 2z_a_. Mean competitive ability of the population equals z_0 _+ 2qz_a_.

### Model 1A: Competition Without Policing

#### Population-genetic dynamics

Setting a_i. _= a_ij _= 0 in eq. [1] (to reflect no policing), the fitness of an individual of genotype j in family-type i is

wij=zijzi.(1−zi.)
 MathType@MTEF@5@5@+=feaafiart1ev1aaatCvAUfKttLearuWrP9MDH5MBPbIqV92AaeXatLxBI9gBaebbnrfifHhDYfgasaacH8akY=wiFfYdH8Gipec8Eeeu0xXdbba9frFj0=OqFfea0dXdd9vqai=hGuQ8kuc9pgc9s8qqaq=dirpe0xb9q8qiLsFr0=vr0=vr0dc8meaabaqaciaacaGaaeqabaqabeGadaaakeaacqWG3bWDdaWgaaWcbaGaemyAaKMaemOAaOgabeaakiabg2da9maalaaabaGaemOEaO3aaSbaaSqaaiabdMgaPjabdQgaQbqabaaakeaacqWG6bGEdaWgaaWcbaGaemyAaKMaeiOla4cabeaaaaGcdaqadaqaaiabigdaXiabgkHiTiabdQha6naaBaaaleaacqWGPbqAcqGGUaGlaeqaaaGccaGLOaGaayzkaaaaaa@41DC@

mean fitness in family i equals

*w*_*i. *_= 1 - *z*_*i*._

and mean fitness across all families is

w¯=1−z¯
 MathType@MTEF@5@5@+=feaafiart1ev1aaatCvAUfKttLearuWrP9MDH5MBPbIqV92AaeXatLxBI9gBaebbnrfifHhDYfgasaacH8akY=wiFfYdH8Gipec8Eeeu0xXdbba9frFj0=OqFfea0dXdd9vqai=hGuQ8kuc9pgc9s8qqaq=dirpe0xb9q8qiLsFr0=vr0=vr0dc8meaabaqaciaacaGaaeqabaqabeGadaaakeaacuWG3bWDgaqeaiabg2da9iabigdaXiabgkHiTiqbdQha6zaaraaaaa@32B3@

We find the frequency of allele *a *after within family selection by taking the product of the frequency of each family type (f_i_), the change in frequency of allele *a *due to selection within this family (Δq_i_), and the mean fitness of this family (w_i_), summing across all family types and dividing by w¯
 MathType@MTEF@5@5@+=feaafiart1ev1aaatCvAUfKttLearuWrP9MDH5MBPbIqV92AaeXatLxBI9gBaebbnrfifHhDYfgasaacH8akY=wiFfYdH8Gipec8Eeeu0xXdbba9frFj0=OqFfea0dXdd9vqai=hGuQ8kuc9pgc9s8qqaq=dirpe0xb9q8qiLsFr0=vr0=vr0dc8meaabaqaciaacaGaaeqabaqabeGadaaakeaacuWG3bWDgaqeaaaa@2E3B@ (see [25] for procedure). Assuming weak selection as in Wade [24,25], we approximate f_i _from Hardy-Weinberg expectations, and thus solutions in text are approximations. In Additional file 1, we present the exact solutions, which differ only slightly from the approximate solutions as also found by Wade [25] for a very different fitness function.

We subtract the frequency of allele a before selection to find the change in q due to selection among and within groups [26-28]

Δqamong=1w¯((∑fiqiwi.)−qw¯)
 MathType@MTEF@5@5@+=feaafiart1ev1aaatCvAUfKttLearuWrP9MDH5MBPbIqV92AaeXatLxBI9gBaebbnrfifHhDYfgasaacH8akY=wiFfYdH8Gipec8Eeeu0xXdbba9frFj0=OqFfea0dXdd9vqai=hGuQ8kuc9pgc9s8qqaq=dirpe0xb9q8qiLsFr0=vr0=vr0dc8meaabaqaciaacaGaaeqabaqabeGadaaakeaacqqHuoarcqWGXbqCdaWgaaWcbaGaemyyaeMaemyBa0Maem4Ba8MaemOBa4Maem4zaCgabeaakiabg2da9maalaaabaGaeGymaedabaGafm4DaCNbaebaaaWaaeWaaeaadaqadaqaamaaqaeabaGaemOzay2aaSbaaSqaaiabdMgaPbqabaGccqWGXbqCdaWgaaWcbaGaemyAaKgabeaakiabdEha3naaBaaaleaacqWGPbqAcqGGUaGlaeqaaaqabeqaniabggHiLdaakiaawIcacaGLPaaacqGHsislcqWGXbqCcuWG3bWDgaqeaaGaayjkaiaawMcaaaaa@4CE9@

Δqwithin=1w¯(∑fiΔqiwi.)
 MathType@MTEF@5@5@+=feaafiart1ev1aaatCvAUfKttLearuWrP9MDH5MBPbIqV92AaeXatLxBI9gBaebbnrfifHhDYfgasaacH8akY=wiFfYdH8Gipec8Eeeu0xXdbba9frFj0=OqFfea0dXdd9vqai=hGuQ8kuc9pgc9s8qqaq=dirpe0xb9q8qiLsFr0=vr0=vr0dc8meaabaqaciaacaGaaeqabaqabeGadaaakeaacqqHuoarcqWGXbqCdaWgaaWcbaGaem4DaCNaemyAaKMaemiDaqNaemiAaGMaemyAaKMaemOBa4gabeaakiabg2da9maalaaabaGaeGymaedabaGafm4DaCNbaebaaaWaaeWaaeaadaaeabqaaiabdAgaMnaaBaaaleaacqWGPbqAaeqaaOGaeuiLdqKaemyCae3aaSbaaSqaaiabdMgaPbqabaGccqWG3bWDdaWgaaWcbaGaemyAaKMaeiOla4cabeaaaeqabeqdcqGHris5aaGccaGLOaGaayzkaaaaaa@4A6A@

Total change in frequency of allele *a *(Δq_total_) is the sum of eqs. [5] and [6]. Values of f_i._, Δq_i._, q_i._, and w_i. _for full and half sib families are presented in Tables 1 and 2, respectively. Table 3 shows mean fitness, as well as Δq among groups, within groups, and total (discussed below).

**Table 1:** Family frequencies, fitnesses, and change in frequency of competitive allele, full-sib families, no policing. From left to right: Family types, frequencies, frequency of offspring genotypes, allele frequency within families, mean offspring competitive intensity, family fitness, and change in frequency of competitive allele due to selection within the family.

Family	Freq	Offspring Genotypes			Family Mean			
		
		AA	Aa	Aa	q_i._	z_i. _= z_iFS_	w_i._	Δq_i._
AA × AA	p^4^	1	---	---	0	z_0 _= z_1FS_	1-z_1FS_	0
AA × Aa	4p^3^q	1/2	1/2	---	1/4	z_0_+z_a_/2 = z_2FS_	1-z_2FS_	z_a_/8z_2FS_
AA × aa	2p^2^q^2^	---	1	---	1/2	z_0_+z_a _= z_3FS_	1-z_3FS_	0
Aa × Aa	4p^2^q^2^	1/4	1/2	1/4	1/2	z_0_+z_a _= z_4FS_	1-z_4FS_	z_a_/4z_4FS_
Aa × aa	4pq^3^	---	1/2	1/2	3/4	z_0_+3z_a_/2 = z_5FS_	1-z_5FS_	z_a_/8z_5FS_
aa × aa	q^4^	---	---	1	1	z_0_+2z_a _= z_6FS_	1-z_6FS_	0

**Table 2:** Family frequencies, fitnesses, and change in frequency of competitive allele, half-sib families, no policing. From left to right: Family types, frequencies, frequency of offspring genotypes, allele frequency within families, mean offspring competitive intensity, family fitness, and change in frequency of competitive allele due to selection within the family.

Fam	Freq	Offspring Genotype			Family Mean			
		
		AA	Aa	aa	qi.	z_i. _= z_iHS_	wi.	Δq_i._
AA	P^2	P	q	---	(q/2)	z_0_+qz_a _= z_1HS_	1-z_1HS_	pqz_a_/2z_1HS_
Aa	2*p*q	P/2	1/2	q/2	(q/2+1/4)	z_0_+z_a_(q+1/2) = z_2HS_	1-z_2HS_	(pqz_a_/2z_2hs_)+(z_a_/8z_2HS_)
aa	q^2	---	p	q	(q/2+1/2)	z_0_+z_a_(q+1) = z_3HS_	1-z_3HS_	pqz_a_/2z_3HS_

**Table 3:** Summary of Model 1A: Evolution of competitive ability with no policing. With no policing, a = a_i. _= a_ij _= 0.

	Half sib	Full sib
Mean fitness (w¯ MathType@MTEF@5@5@+=feaafiart1ev1aaatCvAUfKttLearuWrP9MDH5MBPbIqV92AaeXatLxBI9gBaebbnrfifHhDYfgasaacH8akY=wiFfYdH8Gipec8Eeeu0xXdbba9frFj0=OqFfea0dXdd9vqai=hGuQ8kuc9pgc9s8qqaq=dirpe0xb9q8qiLsFr0=vr0=vr0dc8meaabaqaciaacaGaaeqabaqabeGadaaakeaacuWG3bWDgaqeaaaa@2E3B@)	1 - (*z*_0 _+ 2*qz*_*a*_)	1 - (*z*_0 _+ 2*qz*_*a*_)
Δq within groups (Δq_w_)	pqza(p2z1HS+2pq+12z2HS+q2z3HS−32)/2w¯ MathType@MTEF@5@5@+=feaafiart1ev1aaatCvAUfKttLearuWrP9MDH5MBPbIqV92AaeXatLxBI9gBaebbnrfifHhDYfgasaacH8akY=wiFfYdH8Gipec8Eeeu0xXdbba9frFj0=OqFfea0dXdd9vqai=hGuQ8kuc9pgc9s8qqaq=dirpe0xb9q8qiLsFr0=vr0=vr0dc8meaabaqaciaacaGaaeqabaqabeGadaaakeaacqWGWbaCcqWGXbqCcqWG6bGEdaWgaaWcbaGaemyyaegabeaakmaabmaabaWaaSaaaeaacqWGWbaCdaahaaWcbeqaaiabikdaYaaaaOqaaiabdQha6naaBaaaleaacqaIXaqmcqWGibascqWGtbWuaeqaaaaakiabgUcaRmaalaaabaGaeGOmaiJaemiCaaNaemyCaeNaey4kaSYaaSGaaeaacqaIXaqmaeaacqaIYaGmaaaabaGaemOEaO3aaSbaaSqaaiabikdaYiabdIeaijabdofatbqabaaaaOGaey4kaSYaaSaaaeaacqWGXbqCdaahaaWcbeqaaiabikdaYaaaaOqaaiabdQha6naaBaaaleaacqaIZaWmcqWGibascqWGtbWuaeqaaaaakiabgkHiTmaaliaabaGaeG4mamdabaGaeGOmaidaaaGaayjkaiaawMcaaiabc+caViabikdaYiqbdEha3zaaraaaaa@56D0@	pqza(p2z2FS+2pqz4FS+q2z5FS−1)/2w¯ MathType@MTEF@5@5@+=feaafiart1ev1aaatCvAUfKttLearuWrP9MDH5MBPbIqV92AaeXatLxBI9gBaebbnrfifHhDYfgasaacH8akY=wiFfYdH8Gipec8Eeeu0xXdbba9frFj0=OqFfea0dXdd9vqai=hGuQ8kuc9pgc9s8qqaq=dirpe0xb9q8qiLsFr0=vr0=vr0dc8meaabaqaciaacaGaaeqabaqabeGadaaakeaacqWGWbaCcqWGXbqCcqWG6bGEdaWgaaWcbaGaemyyaegabeaakmaabmaabaWaaSaaaeaacqWGWbaCdaahaaWcbeqaaiabikdaYaaaaOqaaiabdQha6naaBaaaleaacqaIYaGmcqWGgbGrcqWGtbWuaeqaaaaakiabgUcaRmaalaaabaGaeGOmaiJaemiCaaNaemyCaehabaGaemOEaO3aaSbaaSqaaiabisda0iabdAeagjabdofatbqabaaaaOGaey4kaSYaaSaaaeaacqWGXbqCdaahaaWcbeqaaiabikdaYaaaaOqaaiabdQha6naaBaaaleaacqaI1aqncqWGgbGrcqWGtbWuaeqaaaaakiabgkHiTiabigdaXaGaayjkaiaawMcaaiabc+caViabikdaYiqbdEha3zaaraaaaa@52F0@
Δq between groups (Δq_b_)	-*pqz*_*a*_/4w¯ MathType@MTEF@5@5@+=feaafiart1ev1aaatCvAUfKttLearuWrP9MDH5MBPbIqV92AaeXatLxBI9gBaebbnrfifHhDYfgasaacH8akY=wiFfYdH8Gipec8Eeeu0xXdbba9frFj0=OqFfea0dXdd9vqai=hGuQ8kuc9pgc9s8qqaq=dirpe0xb9q8qiLsFr0=vr0=vr0dc8meaabaqaciaacaGaaeqabaqabeGadaaakeaacuWG3bWDgaqeaaaa@2E3B@	-*pqz*_*a*_/2w¯ MathType@MTEF@5@5@+=feaafiart1ev1aaatCvAUfKttLearuWrP9MDH5MBPbIqV92AaeXatLxBI9gBaebbnrfifHhDYfgasaacH8akY=wiFfYdH8Gipec8Eeeu0xXdbba9frFj0=OqFfea0dXdd9vqai=hGuQ8kuc9pgc9s8qqaq=dirpe0xb9q8qiLsFr0=vr0=vr0dc8meaabaqaciaacaGaaeqabaqabeGadaaakeaacuWG3bWDgaqeaaaa@2E3B@
Δq total (Δq_t_)	pqza(p2z1HS+2pq+12z2HS+q2z3HS−2)/2w¯ MathType@MTEF@5@5@+=feaafiart1ev1aaatCvAUfKttLearuWrP9MDH5MBPbIqV92AaeXatLxBI9gBaebbnrfifHhDYfgasaacH8akY=wiFfYdH8Gipec8Eeeu0xXdbba9frFj0=OqFfea0dXdd9vqai=hGuQ8kuc9pgc9s8qqaq=dirpe0xb9q8qiLsFr0=vr0=vr0dc8meaabaqaciaacaGaaeqabaqabeGadaaakeaacqWGWbaCcqWGXbqCcqWG6bGEdaWgaaWcbaGaemyyaegabeaakmaabmaabaWaaSaaaeaacqWGWbaCdaahaaWcbeqaaiabikdaYaaaaOqaaiabdQha6naaBaaaleaacqaIXaqmcqWGibascqWGtbWuaeqaaaaakiabgUcaRmaalaaabaGaeGOmaiJaemiCaaNaemyCaeNaey4kaSYaaSGaaeaacqaIXaqmaeaacqaIYaGmaaaabaGaemOEaO3aaSbaaSqaaiabikdaYiabdIeaijabdofatbqabaaaaOGaey4kaSYaaSaaaeaacqWGXbqCdaahaaWcbeqaaiabikdaYaaaaOqaaiabdQha6naaBaaaleaacqaIZaWmcqWGibascqWGtbWuaeqaaaaakiabgkHiTiabikdaYaGaayjkaiaawMcaaiabc+caViabikdaYiqbdEha3zaaraaaaa@55CA@	pqza(p2z2FS+2pqz4FS+q2z5FS−2)/2w¯ MathType@MTEF@5@5@+=feaafiart1ev1aaatCvAUfKttLearuWrP9MDH5MBPbIqV92AaeXatLxBI9gBaebbnrfifHhDYfgasaacH8akY=wiFfYdH8Gipec8Eeeu0xXdbba9frFj0=OqFfea0dXdd9vqai=hGuQ8kuc9pgc9s8qqaq=dirpe0xb9q8qiLsFr0=vr0=vr0dc8meaabaqaciaacaGaaeqabaqabeGadaaakeaacqWGWbaCcqWGXbqCcqWG6bGEdaWgaaWcbaGaemyyaegabeaakmaabmaabaWaaSaaaeaacqWGWbaCdaahaaWcbeqaaiabikdaYaaaaOqaaiabdQha6naaBaaaleaacqaIYaGmcqWGgbGrcqWGtbWuaeqaaaaakiabgUcaRmaalaaabaGaeGOmaiJaemiCaaNaemyCaehabaGaemOEaO3aaSbaaSqaaiabisda0iabdAeagjabdofatbqabaaaaOGaey4kaSYaaSaaaeaacqWGXbqCdaahaaWcbeqaaiabikdaYaaaaOqaaiabdQha6naaBaaaleaacqaI1aqncqWGgbGrcqWGtbWuaeqaaaaakiabgkHiTiabikdaYaGaayjkaiaawMcaaiabc+caViabikdaYiqbdEha3zaaraaaaa@52F2@

Inputting values from Tables 1 and 2 into eq. [5], we find that selection between families always favors a decrease in competition, regardless of gene frequency, degree of competition, or family type (half sib or full sib). For half sib families, ΔqamongHS=−pqza4w¯
 MathType@MTEF@5@5@+=feaafiart1ev1aaatCvAUfKttLearuWrP9MDH5MBPbIqV92AaeXatLxBI9gBaebbnrfifHhDYfgasaacH8akY=wiFfYdH8Gipec8Eeeu0xXdbba9frFj0=OqFfea0dXdd9vqai=hGuQ8kuc9pgc9s8qqaq=dirpe0xb9q8qiLsFr0=vr0=vr0dc8meaabaqaciaacaGaaeqabaqabeGadaaakeaacqqHuoarcqWGXbqCdaWgaaWcbaGaemyyaeMaemyBa0Maem4Ba8MaemOBa4Maem4zaCMaemisaGKaem4uamfabeaakiabg2da9maalaaabaGaeyOeI0IaemiCaaNaemyCaeNaemOEaO3aaSbaaSqaaiabdggaHbqabaaakeaacqaI0aancuWG3bWDgaqeaaaaaaa@4326@, and is exactly half as strong as among group selection against competition in full sib families ΔqamongFS=−pqza2w¯
 MathType@MTEF@5@5@+=feaafiart1ev1aaatCvAUfKttLearuWrP9MDH5MBPbIqV92AaeXatLxBI9gBaebbnrfifHhDYfgasaacH8akY=wiFfYdH8Gipec8Eeeu0xXdbba9frFj0=OqFfea0dXdd9vqai=hGuQ8kuc9pgc9s8qqaq=dirpe0xb9q8qiLsFr0=vr0=vr0dc8meaabaqaciaacaGaaeqabaqabeGadaaakeaacqqHuoarcqWGXbqCdaWgaaWcbaGaemyyaeMaemyBa0Maem4Ba8MaemOBa4Maem4zaCMaemOrayKaem4uamfabeaakiabg2da9maalaaabaGaeyOeI0IaemiCaaNaemyCaeNaemOEaO3aaSbaaSqaaiabdggaHbqabaaakeaacqaIYaGmcuWG3bWDgaqeaaaaaaa@431E@ as found in many other theoretical studies of kin selection [26-28].

Inputting values from Tables 1 and 2 into eq. [6], we find that comparing results of selection within groups (Table 3, Row 3) is more complicated. Subtracting Δ*q*_*within_FS *_from Δ*q*_*within_HS *_yields pqza2w¯{za(12−q)(p2Z1HSz2FS+2pqZ2HSz4FS+q2Z3HSz5FS)+(1Z2HS−1)/2}
 MathType@MTEF@5@5@+=feaafiart1ev1aaatCvAUfKttLearuWrP9MDH5MBPbIqV92AaeXatLxBI9gBaebbnrfifHhDYfgasaacH8akY=wiFfYdH8Gipec8Eeeu0xXdbba9frFj0=OqFfea0dXdd9vqai=hGuQ8kuc9pgc9s8qqaq=dirpe0xb9q8qiLsFr0=vr0=vr0dc8meaabaqaciaacaGaaeqabaqabeGadaaakeaadaWcaaqaaiabdchaWjabdghaXjabdQha6naaBaaaleaacqWGHbqyaeqaaaGcbaGaeGOmaiJafm4DaCNbaebaaaWaaiWabeaacqWG6bGEdaWgaaWcbaGaemyyaegabeaakmaabmaabaWaaSGaaeaacqaIXaqmaeaacqaIYaGmaaGaeyOeI0IaemyCaehacaGLOaGaayzkaaWaaeWaaeaadaWcaaqaaiabdchaWnaaCaaaleqabaGaeGOmaidaaaGcbaGaemOwaO1aaSbaaSqaaiabigdaXiabdIeaijabdofatbqabaGccqWG6bGEdaWgaaWcbaGaeGOmaiJaemOrayKaem4uamfabeaaaaGccqGHRaWkdaWcaaqaaiabikdaYiabdchaWjabdghaXbqaaiabdQfaAnaaBaaaleaacqaIYaGmcqWGibascqWGtbWuaeqaaOGaemOEaO3aaSbaaSqaaiabisda0iabdAeagjabdofatbqabaaaaOGaey4kaSYaaSaaaeaacqWGXbqCdaahaaWcbeqaaiabikdaYaaaaOqaaiabdQfaAnaaBaaaleaacqaIZaWmcqWGibascqWGtbWuaeqaaOGaemOEaO3aaSbaaSqaaiabiwda1iabdAeagjabdofatbqabaaaaaGccaGLOaGaayzkaaGaey4kaSYaaeWaaeaadaWcaaqaaiabigdaXaqaaiabdQfaAnaaBaaaleaacqaIYaGmcqWGibascqWGtbWuaeqaaaaakiabgkHiTiabigdaXaGaayjkaiaawMcaaiabc+caViabikdaYaGaay5Eaiaaw2haaaaa@7516@. Thus, the difference in the strength of within group selection depends on allele frequency, as well as the level of competition. Specifically, when q < 0.5 and z_2hs _< 1.0, within-group selection favoring competition will be stronger for half-sib families than it is for full-sib families; however, as q rises above 1/2 and z_2hs _rises above 1, within-group selection favoring competition can be stronger for full-sib families than it is for half-sib families. Since z_2HS _cannot exceed 1 in this model, the force of within-group selection can only be stronger in a population of full-sib than half-sib families if the competitive allele is very common.

The formula for total selection on competitive ability is similarly complicated (Table 3, Row 5). Subtracting Δq_total _full-sib from Δq_total _half-sib yields pqza2w¯(za(12−q)(p2Z1HSZ2FS+2pqZ2HSZ4FS+q2Z3HSZ5FS)+12Z2HS)
 MathType@MTEF@5@5@+=feaafiart1ev1aaatCvAUfKttLearuWrP9MDH5MBPbIqV92AaeXatLxBI9gBaebbnrfifHhDYfgasaacH8akY=wiFfYdH8Gipec8Eeeu0xXdbba9frFj0=OqFfea0dXdd9vqai=hGuQ8kuc9pgc9s8qqaq=dirpe0xb9q8qiLsFr0=vr0=vr0dc8meaabaqaciaacaGaaeqabaqabeGadaaakeaadaWcaaqaaiabdchaWjabdghaXjabdQha6naaBaaaleaacqWGHbqyaeqaaaGcbaGaeGOmaiJafm4DaCNbaebaaaWaaeWaaeaacqWG6bGEdaWgaaWcbaGaemyyaegabeaakmaabmaabaWaaSGaaeaacqaIXaqmaeaacqaIYaGmaaGaeyOeI0IaemyCaehacaGLOaGaayzkaaWaaeWaaeaadaWcaaqaaiabdchaWnaaCaaaleqabaGaeGOmaidaaaGcbaGaemOwaO1aaSbaaSqaaiabigdaXiabdIeaijabdofatbqabaGccqWGAbGwdaWgaaWcbaGaeGOmaiJaemOrayKaem4uamfabeaaaaGccqGHRaWkdaWcaaqaaiabikdaYiabdchaWjabdghaXbqaaiabdQfaAnaaBaaaleaacqaIYaGmcqWGibascqWGtbWuaeqaaOGaemOwaO1aaSbaaSqaaiabisda0iabdAeagjabdofatbqabaaaaOGaey4kaSYaaSaaaeaacqWGXbqCdaahaaWcbeqaaiabikdaYaaaaOqaaiabdQfaAnaaBaaaleaacqaIZaWmcqWGibascqWGtbWuaeqaaOGaemOwaO1aaSbaaSqaaiabiwda1iabdAeagjabdofatbqabaaaaaGccaGLOaGaayzkaaGaey4kaSYaaSaaaeaacqaIXaqmaeaacqaIYaGmcqWGAbGwdaWgaaWcbaGaeGOmaiJaemisaGKaem4uamfabeaaaaaakiaawIcacaGLPaaaaaa@6F61@. Since selection on competition is dependent on z_0_, z_a _and q, it is difficult to interpret the effect of family structure on evolution from this result.

#### Solving for optima

**Figure 1:**
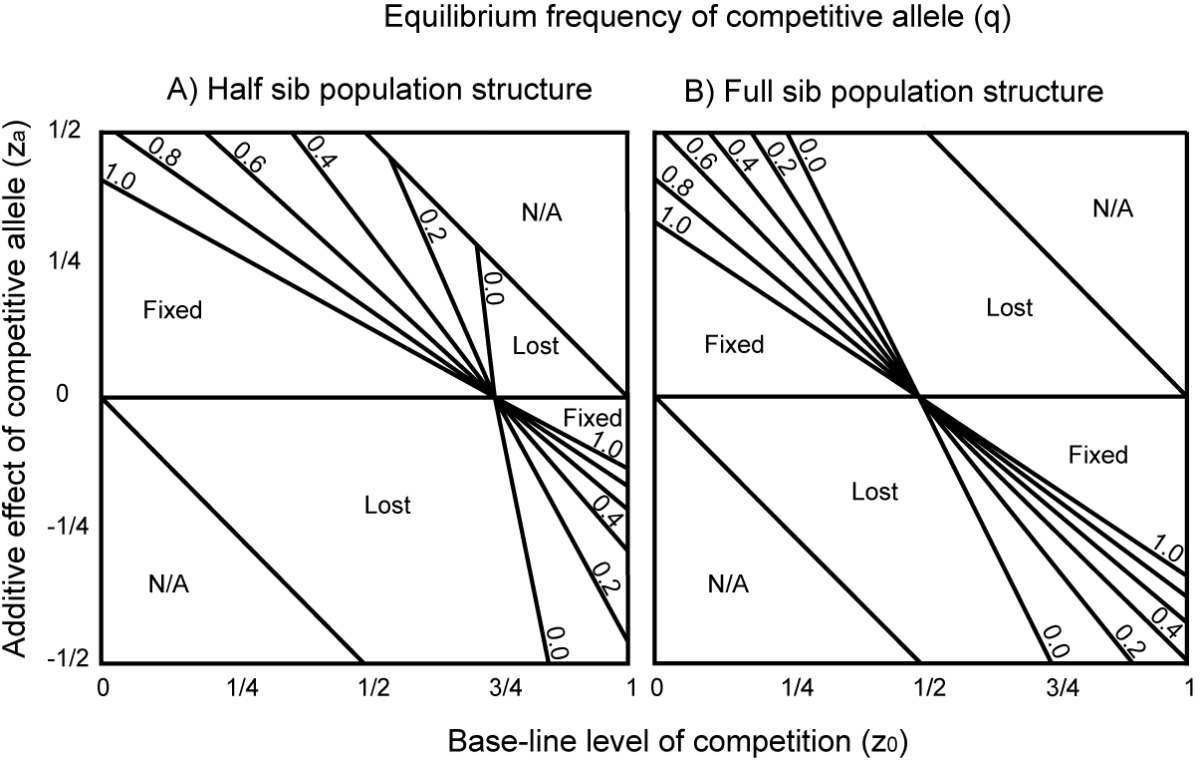
Equilibrium frequency (q*) of a 'competitive' allele. q* as a function of the base-line level of competition (z_0_) and the additive effect of the competitive allele (z_a_). The equilibrium frequency, q* varies continuously from fixed to lost. Lines mark a change in q* of 0.2, and are labeled by numbers to their right.

By solving for the equilibrium value of q* (setting Δq_total _= 0), and comparing results for half and full-sib families (Figure 1), we allow for simple interpretation of the effect of population structure on the evolution of competitive intensity.

**Figure 2:**
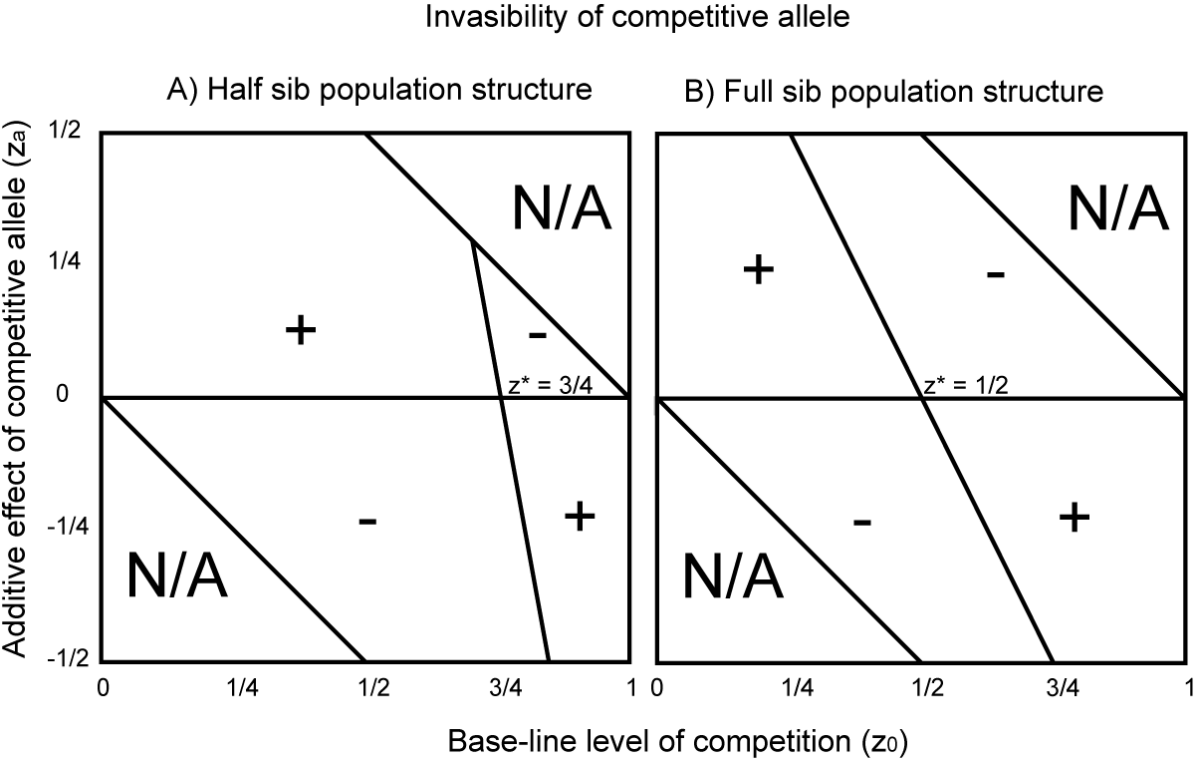
**Invasibility of a 'competitive' allele**. Invasibility of competitive allele with additive effect, z_a_, on its competitive intensity, in a population fixed for z_0 _competitive intensity. The sign, + denotes regions in which the competitive allele can invade when rare, the sign, - denotes regions in which it cannot.

Furthermore, with a few simplifying assumptions we show that this approach provides the same results as Frank [13,14]. We begin with a population fixed for allele p, so that z_.. _= z_0_. We then introduce a rare mutant carrying allele q and find Δq_total _for both half and full sib families. Figures 2a and 2b display the sign of Δq_total _(when q is rare) as a function of z_0 _and z_a _for half and full sib families, respectively. These figures are similar to the pairwise-invasibility-plots of adaptive dynamics [29]; however, rather than presenting z_resident _and z_invader _we present z_0 _(which can be thought of as z_resident_), and z_a _(z_invader _- z_resident_).

We can then obtain the stable level of competition, z*, by setting Δq = 0 and solving for z_0 _as z_a _approaches zero. Let z* represent the invasion equilibrium, the value of z_0 _at which an invader with small levels of z_a _does not change in frequency. This equilibrium equals 3/4 for half sib families and 1/2 for full sib families, coincident with Frank's solution that z* = 1 - r. For both family types, z* is both evolutionarily and continuously stable, meaning that z* cannot be invaded by a rare allele with any nonzero value of z_a _and, reciprocally, that a rare allele entering any monomorphic population will increase in frequency if the allele increases the level of competition and z_0 _is less than z_0_*, or if the allele decreases the level of competition and z_0 _is greater than z_0_*. If the mutant not only invades, but is driven to fixation, then the population becomes monomorphic for a new level of competition, z_0_' which equals z_0 _+ z_a_. Now if a new mutant allele, z_a_, has a small effect on the level of competition and z_0 _+ z_a _individuals are closer to z_0_* than z_0_' this new value of z_a _can spread and now the sum of z_0_' + z_a _becomes the new z_0_. A continuation of this process will ultimately lead to the stable level of competition, z_0_, described above.

#### Population-genetic constrains

However, we are not guaranteed that the evolution of competitive behavior will follow the particular steps outlined above. Of special interest is the observation that, for many combinations of z_0 _and z_a_, there exist a stable level of q and p such that a dimorphism of competitive behavior is maintained (Figures 1A and 1B display equilibrium values of q for half and full sib families, respectively). We can show that such a stable equilibrium is invasible by a mutant with competitive ability closer to z* and resistant to invasion by mutants with competitive ability further from z* (in Additional file 3 we show the derivation of this result for the full-sib model). However, we have not explored the possibility of a new stable intermediate equilibrium, involving 2 or more alleles, in between these boundary analyses. Additionally, it is widely known that complex genetic systems, with dominance, epistasis and pleiotropy can act as constraints, preventing traits from approaching their optima; we have not formally addressed the influence of these factors in the evolution of competitive intensity.

The evolutionary dynamics of competitive ability may also deviate from the description above if competitive ability interacts with another trait that affects the spread of competition. Frank [2,13,14] added policing behavior to the model of competition. Below, we also add a fixed level of policing to this model and examine the evolutionary interaction between these two traits.

### Model 1B: Competition with a Fixed, Nonzero Level of Policing

#### Population-genetic dynamics

With a fixed level of policing (such that a_i. _= a_ij _= a in eq. [1]), the fitness of an individual of genotype j in family-type i changes from eq. [2] to become

wij=(1−zi.(1−a))(a(1−c)+(1−a)zijzi.)
 MathType@MTEF@5@5@+=feaafiart1ev1aaatCvAUfKttLearuWrP9MDH5MBPbIqV92AaeXatLxBI9gBaebbnrfifHhDYfgasaacH8akY=wiFfYdH8Gipec8Eeeu0xXdbba9frFj0=OqFfea0dXdd9vqai=hGuQ8kuc9pgc9s8qqaq=dirpe0xb9q8qiLsFr0=vr0=vr0dc8meaabaqaciaacaGaaeqabaqabeGadaaakeaacqWG3bWDdaWgaaWcbaGaemyAaKMaemOAaOgabeaakiabg2da9maabmaabaGaeGymaeJaeyOeI0IaemOEaO3aaSbaaSqaaiabdMgaPjabc6caUaqabaGcdaqadaqaaiabigdaXiabgkHiTiabdggaHbGaayjkaiaawMcaaaGaayjkaiaawMcaaiabcIcaOiabdggaHnaabmaabaGaeGymaeJaeyOeI0Iaem4yamgacaGLOaGaayzkaaGaey4kaSYaaeWaaeaacqaIXaqmcqGHsislcqWGHbqyaiaawIcacaGLPaaadaWcaaqaaiabdQha6naaBaaaleaacqWGPbqAcqWGQbGAaeqaaaGcbaGaemOEaO3aaSbaaSqaaiabdMgaPjabc6caUaqabaaaaOGaeiykaKcaaa@53D2@

mean fitness in family i equals

*w*_*i. *_= (1 - *z*_*i*._(1 - *a*))(1 - *ca*)

and mean fitness across all families is

w¯=(1−z¯(1−a))(1−ca).
 MathType@MTEF@5@5@+=feaafiart1ev1aaatCvAUfKttLearuWrP9MDH5MBPbIqV92AaeXatLxBI9gBaebbnrfifHhDYfgasaacH8akY=wiFfYdH8Gipec8Eeeu0xXdbba9frFj0=OqFfea0dXdd9vqai=hGuQ8kuc9pgc9s8qqaq=dirpe0xb9q8qiLsFr0=vr0=vr0dc8meaabaqaciaacaGaaeqabaqabeGadaaakeaacuWG3bWDgaqeaiabg2da9maabmaabaGaeGymaeJaeyOeI0IafmOEaONbaebadaqadaqaaiabigdaXiabgkHiTiabdggaHbGaayjkaiaawMcaaaGaayjkaiaawMcaamaabmaabaGaeGymaeJaeyOeI0Iaem4yamMaemyyaegacaGLOaGaayzkaaGaeiOla4caaa@3FD1@

When policing is absent, (i.e., a = 0) eqs. [6-7] reduce to eqs.[2-4]. Tables 4 and 5 show family characteristics including frequencies, fitnesses and allele frequency change for within full and half-sib families, respectively. In Table 6, we show mean fitness, as well as the Δq approximate solutions for half and full-sib families.

**Table 4:** Family frequencies, fitnesses, and change in frequency of competitive allele, full-sib families, fixed policing = a. From left to right: Family types, frequencies, frequency of offspring genotypes, allele frequency within families, mean offspring competitive intensity, family fitness, and change in frequency of competitive allele due to selection within the family.

Family	Freq (f_i._)	Offspring Genotypes			Family Mean			
		
		AA	Aa	aa	q_i._	z_i. _= z_iFS_	w_i._	Δq_i._
AA × AA	p^4^	1	---	---	0	z_0 _= z_1FS_	(1-ca)(1 -z_1FS_(1-a))	0
AA × Aa	4p^3^q	1/2	1/2	---	1/4	z_0_+z_a_/2 = z_2FS_	(1-ca)(1-z_2FS_(1-a))	z_a_(1-a)/(8z_2FS_(1-ac))
AA × aa	2p^2^q^2^	---	1	---	1/2	z_0_+z_a _= z_3FS_	(1-ca)(1-z_3FS_(1-a))	0
Aa × Aa	4p^2^q^2^	1/4	1/2	1/4	1/2	z_0_+z_a _= z_4FS_	(1-ca)(1-z_4FS_(1-a))	z_a_(1-a)/(4z_4FS_(1-ac))
Aa × aa	4pq^3^	---	1/2	1/2	3/4	z_0_+3z_a_/2 = z_5FS_	(1-ca)(1-z_5FS_(1-a))	z_a_(1-a)/(8z_5FS_(1-ac))
aa × aa	q^4^	---	---	1	1	z_0_+2z_a _= z_6FS_	(1-ca)(1-z_6FS_(1-a))	0

**Table 5:** Family frequencies, fitnesses, and change in frequency of competitive allele, half-sib families, fixed policing = a. From left to right: Family types, frequencies, frequency of offspring genotypes, allele frequency within families, mean offspring competitive intensity, family fitness, and change in frequency of competitive allele due to selection within the family.

Fam	Freq	Offspring Genotype			Family Mean			
		
		AA	Aa	aa	qi.	z_i _= z_iHS_	wi.	Δqi.
AA	p^2	p	q	---	(q/2)	z_0_+qza = z_1HS_	(1-ca)(1-z_1HS_(1-a))	pqz_a_(1-a)/(2z_1HS_(1-ac))
Aa	2*p*q	p/2	1/2	q/2	(q/2+1/4)	z_0_+za(q+1/2) = z_2HS_	(1-ca)(1-z_2HS_(1-a))	((za(1-a))/(z_2HS_(1-ac)))(1/8+pq/2)
aa	q^2	---	p	q	(q/2+1/2)	z_0_+za(q+1) = z_3HS_	(1-ca)(1-z_3HS_(1-a))	pqz_a_(1-a)/(2z_3HS_(1-ac))

**Table 6:** Summary of Model 1A: Evolution of competitive ability with fixed policing = a = a_i _= a_ij_

	Half sib	Full sib
Mean fitness (w¯ MathType@MTEF@5@5@+=feaafiart1ev1aaatCvAUfKttLearuWrP9MDH5MBPbIqV92AaeXatLxBI9gBaebbnrfifHhDYfgasaacH8akY=wiFfYdH8Gipec8Eeeu0xXdbba9frFj0=OqFfea0dXdd9vqai=hGuQ8kuc9pgc9s8qqaq=dirpe0xb9q8qiLsFr0=vr0=vr0dc8meaabaqaciaacaGaaeqabaqabeGadaaakeaacuWG3bWDgaqeaaaa@2E3B@)	(1 - *z*(1 - *a*_._))(1 - *ca*_._)	(1 - *z*(1 - *a*_._))(1 - *ca*_._)
Δq within groups (Δq_w_)	pqza(1−a.)(p2z1HS+2pq+12z2HS+q2z3HS−3(1−a)2)/(2w¯FS) MathType@MTEF@5@5@+=feaafiart1ev1aaatCvAUfKttLearuWrP9MDH5MBPbIqV92AaeXatLxBI9gBaebbnrfifHhDYfgasaacH8akY=wiFfYdH8Gipec8Eeeu0xXdbba9frFj0=OqFfea0dXdd9vqai=hGuQ8kuc9pgc9s8qqaq=dirpe0xb9q8qiLsFr0=vr0=vr0dc8meaabaqaciaacaGaaeqabaqabeGadaaakeaacqWGWbaCcqWGXbqCcqWG6bGEdaWgaaWcbaGaemyyaegabeaakmaabmaabaGaeGymaeJaeyOeI0Iaemyyae2aaSbaaSqaaiabc6caUaqabaaakiaawIcacaGLPaaadaqadaqaamaalaaabaGaemiCaa3aaWbaaSqabeaacqaIYaGmaaaakeaacqWG6bGEdaWgaaWcbaGaeGymaeJaemisaGKaem4uamfabeaaaaGccqGHRaWkdaWcaaqaaiabikdaYiabdchaWjabdghaXjabgUcaRmaaliaabaGaeGymaedabaGaeGOmaidaaaqaaiabdQha6naaBaaaleaacqaIYaGmcqWGibascqWGtbWuaeqaaaaakiabgUcaRmaalaaabaGaemyCae3aaWbaaSqabeaacqaIYaGmaaaakeaacqWG6bGEdaWgaaWcbaGaeG4mamJaemisaGKaem4uamfabeaaaaGccqGHsisldaWcaaqaaiabiodaZmaabmaabaGaeGymaeJaeyOeI0IaemyyaegacaGLOaGaayzkaaaabaGaeGOmaidaaaGaayjkaiaawMcaaiabc+caVmaabmaabaGaeGOmaiJafm4DaCNbaebadaWgaaWcbaGaemOrayKaem4uamfabeaaaOGaayjkaiaawMcaaaaa@654D@	pqza(1−a.)(p2z2FS+2pqz4FS+q2z4FS+a−1)/(2w¯FS) MathType@MTEF@5@5@+=feaafiart1ev1aaatCvAUfKttLearuWrP9MDH5MBPbIqV92AaeXatLxBI9gBaebbnrfifHhDYfgasaacH8akY=wiFfYdH8Gipec8Eeeu0xXdbba9frFj0=OqFfea0dXdd9vqai=hGuQ8kuc9pgc9s8qqaq=dirpe0xb9q8qiLsFr0=vr0=vr0dc8meaabaqaciaacaGaaeqabaqabeGadaaakeaacqWGWbaCcqWGXbqCcqWG6bGEdaWgaaWcbaGaemyyaegabeaakmaabmaabaGaeGymaeJaeyOeI0Iaemyyae2aaSbaaSqaaiabc6caUaqabaaakiaawIcacaGLPaaadaqadaqaamaalaaabaGaemiCaa3aaWbaaSqabeaacqaIYaGmaaaakeaacqWG6bGEdaWgaaWcbaGaeGOmaiJaemOrayKaem4uamfabeaaaaGccqGHRaWkdaWcaaqaaiabikdaYiabdchaWjabdghaXbqaaiabdQha6naaBaaaleaacqaI0aancqWGgbGrcqWGtbWuaeqaaaaakiabgUcaRmaalaaabaGaemyCae3aaWbaaSqabeaacqaIYaGmaaaakeaacqWG6bGEdaWgaaWcbaGaeGinaqJaemOrayKaem4uamfabeaaaaGccqGHRaWkcqWGHbqycqGHsislcqaIXaqmaiaawIcacaGLPaaacqGGVaWldaqadaqaaiabikdaYiqbdEha3zaaraWaaSbaaSqaaiabdAeagjabdofatbqabaaakiaawIcacaGLPaaaaaa@5EE9@
Δq between groups (Δq_b_)	-*pqz*_*a*._(1 - *a*_._)(1 - *a_._c*)/(4w¯ MathType@MTEF@5@5@+=feaafiart1ev1aaatCvAUfKttLearuWrP9MDH5MBPbIqV92AaeXatLxBI9gBaebbnrfifHhDYfgasaacH8akY=wiFfYdH8Gipec8Eeeu0xXdbba9frFj0=OqFfea0dXdd9vqai=hGuQ8kuc9pgc9s8qqaq=dirpe0xb9q8qiLsFr0=vr0=vr0dc8meaabaqaciaacaGaaeqabaqabeGadaaakeaacuWG3bWDgaqeaaaa@2E3B@_*HS*_)	-*pqz*_*a*._(1 - *a*_._)(1 - *a_._c*)/(2w¯ MathType@MTEF@5@5@+=feaafiart1ev1aaatCvAUfKttLearuWrP9MDH5MBPbIqV92AaeXatLxBI9gBaebbnrfifHhDYfgasaacH8akY=wiFfYdH8Gipec8Eeeu0xXdbba9frFj0=OqFfea0dXdd9vqai=hGuQ8kuc9pgc9s8qqaq=dirpe0xb9q8qiLsFr0=vr0=vr0dc8meaabaqaciaacaGaaeqabaqabeGadaaakeaacuWG3bWDgaqeaaaa@2E3B@_*FS*_)
Δq total (Δq_t_)	pqza(1−a.)(p2z1HS+2pq+12z2HS+q2z3HS+a(3+c)2−2)/(2w¯HS) MathType@MTEF@5@5@+=feaafiart1ev1aaatCvAUfKttLearuWrP9MDH5MBPbIqV92AaeXatLxBI9gBaebbnrfifHhDYfgasaacH8akY=wiFfYdH8Gipec8Eeeu0xXdbba9frFj0=OqFfea0dXdd9vqai=hGuQ8kuc9pgc9s8qqaq=dirpe0xb9q8qiLsFr0=vr0=vr0dc8meaabaqaciaacaGaaeqabaqabeGadaaakeaacqWGWbaCcqWGXbqCcqWG6bGEdaWgaaWcbaGaemyyaegabeaakmaabmaabaGaeGymaeJaeyOeI0Iaemyyae2aaSbaaSqaaiabc6caUaqabaaakiaawIcacaGLPaaadaqadaqaamaalaaabaGaemiCaa3aaWbaaSqabeaacqaIYaGmaaaakeaacqWG6bGEdaWgaaWcbaGaeGymaeJaemisaGKaem4uamfabeaaaaGccqGHRaWkdaWcaaqaaiabikdaYiabdchaWjabdghaXjabgUcaRmaaliaabaGaeGymaedabaGaeGOmaidaaaqaaiabdQha6naaBaaaleaacqaIYaGmcqWGibascqWGtbWuaeqaaaaakiabgUcaRmaalaaabaGaemyCae3aaWbaaSqabeaacqaIYaGmaaaakeaacqWG6bGEdaWgaaWcbaGaeG4mamJaemisaGKaem4uamfabeaaaaGccqGHRaWkdaWcaaqaaiabdggaHnaabmaabaGaeG4mamJaey4kaSIaem4yamgacaGLOaGaayzkaaaabaGaeGOmaidaaiabgkHiTiabikdaYaGaayjkaiaawMcaaiabc+caVmaabmaabaGaeGOmaiJafm4DaCNbaebadaWgaaWcbaGaemisaGKaem4uamfabeaaaOGaayjkaiaawMcaaaaa@6779@	pqza(1−a.)(p2z2FS+2pqz4FS+q2z5FS+a(1+c)−2)/(2w¯FS) MathType@MTEF@5@5@+=feaafiart1ev1aaatCvAUfKttLearuWrP9MDH5MBPbIqV92AaeXatLxBI9gBaebbnrfifHhDYfgasaacH8akY=wiFfYdH8Gipec8Eeeu0xXdbba9frFj0=OqFfea0dXdd9vqai=hGuQ8kuc9pgc9s8qqaq=dirpe0xb9q8qiLsFr0=vr0=vr0dc8meaabaqaciaacaGaaeqabaqabeGadaaakeaacqWGWbaCcqWGXbqCcqWG6bGEdaWgaaWcbaGaemyyaegabeaakmaabmaabaGaeGymaeJaeyOeI0Iaemyyae2aaSbaaSqaaiabc6caUaqabaaakiaawIcacaGLPaaadaqadaqaamaalaaabaGaemiCaa3aaWbaaSqabeaacqaIYaGmaaaakeaacqWG6bGEdaWgaaWcbaGaeGOmaiJaemOrayKaem4uamfabeaaaaGccqGHRaWkdaWcaaqaaiabikdaYiabdchaWjabdghaXbqaaiabdQha6naaBaaaleaacqaI0aancqWGgbGrcqWGtbWuaeqaaaaakiabgUcaRmaalaaabaGaemyCae3aaWbaaSqabeaacqaIYaGmaaaakeaacqWG6bGEdaWgaaWcbaGaeGynauJaemOrayKaem4uamfabeaaaaGccqGHRaWkcqWGHbqydaqadaqaaiabigdaXiabgUcaRiabdogaJbGaayjkaiaawMcaaiabgkHiTiabikdaYaGaayjkaiaawMcaaiabc+caVmaabmaabaGaeGOmaiJafm4DaCNbaebadaWgaaWcbaGaemOrayKaem4uamfabeaaaOGaayjkaiaawMcaaaaa@6397@

#### Solving for optima

From the Δq total expressions in Table 6 we derived z_0_* for half and full-sib families. Frank ([13] – figure 1B) shows that, when selection favors any level of policing, (1) policing effort (a) will approach 1, and (2) the level of competition will evolve to 1−rr(1−c)
 MathType@MTEF@5@5@+=feaafiart1ev1aaatCvAUfKttLearuWrP9MDH5MBPbIqV92AaeXatLxBI9gBaebbnrfifHhDYfgasaacH8akY=wiFfYdH8Gipec8Eeeu0xXdbba9frFj0=OqFfea0dXdd9vqai=hGuQ8kuc9pgc9s8qqaq=dirpe0xb9q8qiLsFr0=vr0=vr0dc8meaabaqaciaacaGaaeqabaqabeGadaaakeaadaWcaaqaaiabigdaXiabgkHiTiabdkhaYbqaaiabdkhaYnaabmaabaGaeGymaeJaeyOeI0Iaem4yamgacaGLOaGaayzkaaaaaaaa@3628@. Here we address prediction 2 by inserting the values, r = 1/2 and 3/4 for full and half sib families, respectively, into Frank's prediction and comparing it to our own derivation of z_0_*. As a → 1, we find that the two approaches provide equivalent solutions, namely, zHS(a==1)∗=31−c
 MathType@MTEF@5@5@+=feaafiart1ev1aaatCvAUfKttLearuWrP9MDH5MBPbIqV92AaeXatLxBI9gBaebbnrfifHhDYfgasaacH8akY=wiFfYdH8Gipec8Eeeu0xXdbba9frFj0=OqFfea0dXdd9vqai=hGuQ8kuc9pgc9s8qqaq=dirpe0xb9q8qiLsFr0=vr0=vr0dc8meaabaqaciaacaGaaeqabaqabeGadaaakeaacqWG6bGEdaqhaaWcbaGaemisaGKaem4uam1aaeWaaeaacqWGHbqycqGH9aqpcqGH9aqpcqaIXaqmaiaawIcacaGLPaaaaeaacqGHxiIkaaGccqGH9aqpdaWcaaqaaiabiodaZaqaaiabigdaXiabgkHiTiabdogaJbaaaaa@3C9D@ and zFS(a==1)∗=11−c
 MathType@MTEF@5@5@+=feaafiart1ev1aaatCvAUfKttLearuWrP9MDH5MBPbIqV92AaeXatLxBI9gBaebbnrfifHhDYfgasaacH8akY=wiFfYdH8Gipec8Eeeu0xXdbba9frFj0=OqFfea0dXdd9vqai=hGuQ8kuc9pgc9s8qqaq=dirpe0xb9q8qiLsFr0=vr0=vr0dc8meaabaqaciaacaGaaeqabaqabeGadaaakeaacqWG6bGEdaqhaaWcbaGaemOrayKaem4uam1aaeWaaeaacqWGHbqycqGH9aqpcqGH9aqpcqaIXaqmaiaawIcacaGLPaaaaeaacqGHxiIkaaGccqGH9aqpdaWcaaqaaiabigdaXaqaaiabigdaXiabgkHiTiabdogaJbaaaaa@3C95@. Intriguingly, both solutions result in values of z* > 1, which, in the absence of policing, would result in *negative *fitnesses (see eqs. [2-4]). Values of z* in excess of 1 are only possible with some level of policing already present in the population, so that no families or individuals suffer from w < 0. Frank ([14] p. 1166) explained this result as follows:

"The high competitiveness in a policing situation is no different from high internal pressure in a fish that lives at great depth. The fish brought to the surface explodes; intense competition and avoidance of repressive policing cause chaos when the same amount of energy is devoted to competition in the absence of repressive policing. "

Although Frank's and our approaches provide equivalent solutions for the level of competition in half and full-sib structured populations with complete policing, some information is lost in this comparison. Frank's approach allows for the derivation of z* with any quantitative value of r, while we have only solved for two specific values of relatedness, r = 1/2 and r = 1/4. By contrast, our model provides a prediction for the optimal level of competition when the level of policing (a) lies between the two stable solutions (1 and 0). Thus, the two derivations of z* provide complimentary solutions to the evolution of competitive ability in a police state.

### Summary of Model 1: The Evolution of Competitive Ability

We apply a family selection approach to the problem of the evolution of interference competition. In the long term, without 'policing,' we find that mean competitive ability will approach a value of 1/2 for full-sib and 1/4 for half-sib families, in agreement with previous research [13]. However, we also discovered a plane of stable equlibria between competitive specialists and a less competitive class. When a fixed level of policing behavior is added to this model, the regions of stable equilibria are reduced and a sharp increase in competitive ability is favored. In some sense, although policing limits the expression of competitive ability, the innate tendency to compete is enhanced beyond that able to evolve in the absence of policing: policing permits the evolution of more extreme interference competition.

### Model 2: The Evolution of Policing with a Fixed Level of Competition

We now examine the evolution of policing behavior in a population with a fixed level of competition, assuming that an individual's level of policing is controlled by one locus with two alleles with additive affects. Let allele B occur in frequency t and the alternative allele, b, in frequency u = (1 - t), so that, after random mating, BB, Bb, and bb individuals are in frequencies t^2^, 2tu, and u^2^, respectively. Let BB individuals have a baseline level of policing denoted by a_0_. Adding a b allele to the genotype increases policing effort by an amount, a_a_. Thus Bb individuals police at the level a_0 _+ a_a_, while bb individuals police at the level, a_0 _+ 2a_a_. Mean policing effort of the population equals a_0_+2ua_a_.

#### Population-genetic dynamics

In this model, policing decreases the benefit of competition to within-group fitness, and decreases the deleterious effect of competition on between-group fitness. Following [1], and setting z = z_ij _= z_i. _= z_.. _to represent a fixed level of competition, the fitness of the j^th ^individual in the i^th ^group equals

*w*_*ij *_= (1 - *ca*_*ij*_)(1 - *z*(1 - *a*_*i*._))

the mean fitness of group i equals

*w*_*i*. _= (1 - *ca*_*i*._)(1 - *z*(1 - *a*_*i*._))

and population mean fitness equals

w¯=(1−cai.)(1−z(1−ai.))−σa_AF2
 MathType@MTEF@5@5@+=feaafiart1ev1aaatCvAUfKttLearuWrP9MDH5MBPbIqV92AaeXatLxBI9gBaebbnrfifHhDYfgasaacH8akY=wiFfYdH8Gipec8Eeeu0xXdbba9frFj0=OqFfea0dXdd9vqai=hGuQ8kuc9pgc9s8qqaq=dirpe0xb9q8qiLsFr0=vr0=vr0dc8meaabaqaciaacaGaaeqabaqabeGadaaakeaacuWG3bWDgaqeaiabg2da9maabmaabaGaeGymaeJaeyOeI0Iaem4yamMaemyyae2aaSbaaSqaaiabdMgaPjabc6caUaqabaaakiaawIcacaGLPaaadaqadaqaaiabigdaXiabgkHiTiabdQha6naabmaabaGaeGymaeJaeyOeI0Iaemyyae2aaSbaaSqaaiabdMgaPjabc6caUaqabaaakiaawIcacaGLPaaaaiaawIcacaGLPaaacqGHsisliiGacqWFdpWCdaqhaaWcbaGaemyyaeMaei4xa8LaemyqaeKaemOrayeabaGaeGOmaidaaaaa@4C46@

where c and a represent the cost and degree of policing, respectively, *σ*^2^_a_AF _represents the among-family variance in policing effort. For both half and full sib families, we find the frequency of allele b after selection among and within families following eqs. [5] and [6] respectively, maintaining the same major assumptions, but substituting u for q. Values of f_i._, Δu_i._, u_i._, and w_i. _for full and half-sib family-structured populations are presented in Tables 7 and 8, respectively.

**Table 7:** Family frequencies, fitnesses, and change in frequency of policing allele, full-sib families, fixed competition = z. From left to right: Family types, frequencies, frequency of offspring genotypes, allele frequency within families, mean offspring policing level, family fitness, and change in frequency of competitive allele due to selection within the family.

Family	Freq	Offspring Genotypes			Family Mean			
		
		BB	Bb	bb	u_i_	a_i _= a_iFS_	wi	Δu_i._
BB × BB	t^4^	--	---	---	0	a_0 _= a_1FS_	(1-ca_1FS_)(1-z(1-a_1FS_))	0
BB × Bb	4t^3^u	1/2	1/2	---	1/4	a_0_+a_a_/2 = a_2FS_	(1-ca_2FS_)(1-z(1-a_2FS_))	-ca_a_/(8(1-ca_2FS_))
BB × bb	2t^2^u^2^	---	1	---	1/2	a_0_+a_a _= a_3FS_	(1-ca_3FS_)(1-z(1-a_3FS_))	0
Bb × Bb	4t^2^u^2^	1/4	1/2	1/4	1/2	a_0_+a_a _= a_4FS_	(1-ca_4FS_)(1-z(1-a_4FS_))	-ca_a_/(4(1-ca_4FS_))
Bb × bb	4tu^3^	---	1/2	1/2	3/4	a_0_+3a_a_/2 = a_5FS_	(1-ca_5FS_)(1-z(1-a_5FS_))	-ca_a_/(8(1-ca_5FS_))
bb × bb	u^4^	---	---	1	1	a_0_+2a_a _= a_6FS_	(1-ca_6FS_)(1-z(1-a_6FS_))	0

**Table 8:** Family frequencies, fitnesses, and change in frequency of policing allele, half-sib families, fixed competition = z. From left to right: Family types, frequencies, frequency of offspring genotypes, allele frequency within families, mean offspring policing level, family fitness, and change in frequency of competitive allele due to selection within the family.

Family	Freq	Offspring Genotypes			Family Mean			
		
		B	Bb	bb	u_i_	a_i _= a_iHS_	w_i._	Δu_i_
BB	t^2^	t	u	---	(u/2)	a_0_+uaa = a_2HS_	(1-ca_1HS_)(1-z(1-a_1HS_))	-utca_a_/2(1-ca_1HS_)
Bb	2tu	t/2	1/2	u/2	(u/2 + 1/4)	a_0_+aa(u+1/2) = a_2HS_	(1-ca_2HS_)(1-z(l-a_2HS_))	-ca_a_/(1 -ca_2HS_)(1/8+u*t/2)
bb	u^2^	---	t	u	(u/2+1/2)	a_0_+aa(u+1) = a_3HS_	(1-ca_3HS_)(1-z(1-a_3HS_))	-utca_a_/2(1-ca_3HS_)

We find the surprising result that, unlike the model of competition, with all else being equal, the w¯
 MathType@MTEF@5@5@+=feaafiart1ev1aaatCvAUfKttLearuWrP9MDH5MBPbIqV92AaeXatLxBI9gBaebbnrfifHhDYfgasaacH8akY=wiFfYdH8Gipec8Eeeu0xXdbba9frFj0=OqFfea0dXdd9vqai=hGuQ8kuc9pgc9s8qqaq=dirpe0xb9q8qiLsFr0=vr0=vr0dc8meaabaqaciaacaGaaeqabaqabeGadaaakeaacuWG3bWDgaqeaaaa@2E3B@_*HS *_≠ w¯
 MathType@MTEF@5@5@+=feaafiart1ev1aaatCvAUfKttLearuWrP9MDH5MBPbIqV92AaeXatLxBI9gBaebbnrfifHhDYfgasaacH8akY=wiFfYdH8Gipec8Eeeu0xXdbba9frFj0=OqFfea0dXdd9vqai=hGuQ8kuc9pgc9s8qqaq=dirpe0xb9q8qiLsFr0=vr0=vr0dc8meaabaqaciaacaGaaeqabaqabeGadaaakeaacuWG3bWDgaqeaaaa@2E3B@_*FS*_. This result follows from the emergence of ai.2
 MathType@MTEF@5@5@+=feaafiart1ev1aaatCvAUfKttLearuWrP9MDH5MBPbIqV92AaeXatLxBI9gBaebbnrfifHhDYfgasaacH8akY=wiFfYdH8Gipec8Eeeu0xXdbba9frFj0=OqFfea0dXdd9vqai=hGuQ8kuc9pgc9s8qqaq=dirpe0xb9q8qiLsFr0=vr0=vr0dc8meaabaqaciaacaGaaeqabaqabeGadaaakeaacqWGHbqydaqhaaWcbaGaemyAaKMaeiOla4cabaGaeGOmaidaaaaa@3155@ in the derivation of *w*_*i*. _(eq. [8]) – in replacing *a*_*i *_with a¯
 MathType@MTEF@5@5@+=feaafiart1ev1aaatCvAUfKttLearuWrP9MDH5MBPbIqV92AaeXatLxBI9gBaebbnrfifHhDYfgasaacH8akY=wiFfYdH8Gipec8Eeeu0xXdbba9frFj0=OqFfea0dXdd9vqai=hGuQ8kuc9pgc9s8qqaq=dirpe0xb9q8qiLsFr0=vr0=vr0dc8meaabaqaciaacaGaaeqabaqabeGadaaakeaacuWGHbqygaqeaaaa@2E0F@ in eq. [9] we substitute the square of the mean level of policing for the mean of the squared level of policing. We therefore subtract the difference between these values (i.e. the variance in policing) multiplied by c and z. Since multiple mating decreases the variance among maternal families, w¯
 MathType@MTEF@5@5@+=feaafiart1ev1aaatCvAUfKttLearuWrP9MDH5MBPbIqV92AaeXatLxBI9gBaebbnrfifHhDYfgasaacH8akY=wiFfYdH8Gipec8Eeeu0xXdbba9frFj0=OqFfea0dXdd9vqai=hGuQ8kuc9pgc9s8qqaq=dirpe0xb9q8qiLsFr0=vr0=vr0dc8meaabaqaciaacaGaaeqabaqabeGadaaakeaacuWG3bWDgaqeaaaa@2E3B@_*HS *_≥ w¯
 MathType@MTEF@5@5@+=feaafiart1ev1aaatCvAUfKttLearuWrP9MDH5MBPbIqV92AaeXatLxBI9gBaebbnrfifHhDYfgasaacH8akY=wiFfYdH8Gipec8Eeeu0xXdbba9frFj0=OqFfea0dXdd9vqai=hGuQ8kuc9pgc9s8qqaq=dirpe0xb9q8qiLsFr0=vr0=vr0dc8meaabaqaciaacaGaaeqabaqabeGadaaakeaacuWG3bWDgaqeaaaa@2E3B@_*FS*_. The difference between w¯
 MathType@MTEF@5@5@+=feaafiart1ev1aaatCvAUfKttLearuWrP9MDH5MBPbIqV92AaeXatLxBI9gBaebbnrfifHhDYfgasaacH8akY=wiFfYdH8Gipec8Eeeu0xXdbba9frFj0=OqFfea0dXdd9vqai=hGuQ8kuc9pgc9s8qqaq=dirpe0xb9q8qiLsFr0=vr0=vr0dc8meaabaqaciaacaGaaeqabaqabeGadaaakeaacuWG3bWDgaqeaaaa@2E3B@_*HS *_and w¯
 MathType@MTEF@5@5@+=feaafiart1ev1aaatCvAUfKttLearuWrP9MDH5MBPbIqV92AaeXatLxBI9gBaebbnrfifHhDYfgasaacH8akY=wiFfYdH8Gipec8Eeeu0xXdbba9frFj0=OqFfea0dXdd9vqai=hGuQ8kuc9pgc9s8qqaq=dirpe0xb9q8qiLsFr0=vr0=vr0dc8meaabaqaciaacaGaaeqabaqabeGadaaakeaacuWG3bWDgaqeaaaa@2E3B@_*FS *_equals zctuaa22
 MathType@MTEF@5@5@+=feaafiart1ev1aaatCvAUfKttLearuWrP9MDH5MBPbIqV92AaeXatLxBI9gBaebbnrfifHhDYfgasaacH8akY=wiFfYdH8Gipec8Eeeu0xXdbba9frFj0=OqFfea0dXdd9vqai=hGuQ8kuc9pgc9s8qqaq=dirpe0xb9q8qiLsFr0=vr0=vr0dc8meaabaqaciaacaGaaeqabaqabeGadaaakeaadaWcaaqaaiabdQha6jabdogaJjabdsha0jabdwha1jabdggaHnaaDaaaleaacqWGHbqyaeaacqaIYaGmaaaakeaacqaIYaGmaaaaaa@371D@, which is zc times the difference in among-family variance in policing effort for half and full-sib families (Table 9).

**Table 9:** Summary of Model 2: Evolution of policing effort with fixed levels of competition = z.

	Half sib	Full sib
Among-family variance in policing (*σ*^2^_a_AF_)	*tua*_*a*_^2^/2	*tua*_*a*_^2^
Mean fitness (***W***)	(1 - *c*a¯ MathType@MTEF@5@5@+=feaafiart1ev1aaatCvAUfKttLearuWrP9MDH5MBPbIqV92AaeXatLxBI9gBaebbnrfifHhDYfgasaacH8akY=wiFfYdH8Gipec8Eeeu0xXdbba9frFj0=OqFfea0dXdd9vqai=hGuQ8kuc9pgc9s8qqaq=dirpe0xb9q8qiLsFr0=vr0=vr0dc8meaabaqaciaacaGaaeqabaqabeGadaaakeaacuWGHbqygaqeaaaa@2E0F@)(1 - *z*(1 - a¯ MathType@MTEF@5@5@+=feaafiart1ev1aaatCvAUfKttLearuWrP9MDH5MBPbIqV92AaeXatLxBI9gBaebbnrfifHhDYfgasaacH8akY=wiFfYdH8Gipec8Eeeu0xXdbba9frFj0=OqFfea0dXdd9vqai=hGuQ8kuc9pgc9s8qqaq=dirpe0xb9q8qiLsFr0=vr0=vr0dc8meaabaqaciaacaGaaeqabaqabeGadaaakeaacuWGHbqygaqeaaaa@2E0F@)) - *czσ*^2^_*a_AF_HS*_	(1 - *c*a¯ MathType@MTEF@5@5@+=feaafiart1ev1aaatCvAUfKttLearuWrP9MDH5MBPbIqV92AaeXatLxBI9gBaebbnrfifHhDYfgasaacH8akY=wiFfYdH8Gipec8Eeeu0xXdbba9frFj0=OqFfea0dXdd9vqai=hGuQ8kuc9pgc9s8qqaq=dirpe0xb9q8qiLsFr0=vr0=vr0dc8meaabaqaciaacaGaaeqabaqabeGadaaakeaacuWGHbqygaqeaaaa@2E0F@)(1 - *z*(1 - a¯ MathType@MTEF@5@5@+=feaafiart1ev1aaatCvAUfKttLearuWrP9MDH5MBPbIqV92AaeXatLxBI9gBaebbnrfifHhDYfgasaacH8akY=wiFfYdH8Gipec8Eeeu0xXdbba9frFj0=OqFfea0dXdd9vqai=hGuQ8kuc9pgc9s8qqaq=dirpe0xb9q8qiLsFr0=vr0=vr0dc8meaabaqaciaacaGaaeqabaqabeGadaaakeaacuWGHbqygaqeaaaa@2E0F@)) - *czσ*^2^_*a_AF_FS*_
Δu within groups (Δu_w_)	−aactu4w¯HS(3−z(3−(2a¯+a2HS))) MathType@MTEF@5@5@+=feaafiart1ev1aaatCvAUfKttLearuWrP9MDH5MBPbIqV92AaeXatLxBI9gBaebbnrfifHhDYfgasaacH8akY=wiFfYdH8Gipec8Eeeu0xXdbba9frFj0=OqFfea0dXdd9vqai=hGuQ8kuc9pgc9s8qqaq=dirpe0xb9q8qiLsFr0=vr0=vr0dc8meaabaqaciaacaGaaeqabaqabeGadaaakeaadaWcaaqaaiabgkHiTiabdggaHnaaBaaaleaacqWGHbqyaeqaaOGaem4yamMaemiDaqNaemyDauhabaGaeGinaqJafm4DaCNbaebadaWgaaWcbaGaemisaGKaem4uamfabeaaaaGcdaqadaqaaiabiodaZiabgkHiTiabdQha6naabmaabaGaeG4mamJaeyOeI0YaaeWaaeaacqaIYaGmcuWGHbqygaqeaiabgUcaRiabdggaHnaaBaaaleaacqaIYaGmcqWGibascqWGtbWuaeqaaaGccaGLOaGaayzkaaaacaGLOaGaayzkaaaacaGLOaGaayzkaaaaaa@4B77@	−aactu2w¯FS(1−z(1−(a¯+a4FS)/2)) MathType@MTEF@5@5@+=feaafiart1ev1aaatCvAUfKttLearuWrP9MDH5MBPbIqV92AaeXatLxBI9gBaebbnrfifHhDYfgasaacH8akY=wiFfYdH8Gipec8Eeeu0xXdbba9frFj0=OqFfea0dXdd9vqai=hGuQ8kuc9pgc9s8qqaq=dirpe0xb9q8qiLsFr0=vr0=vr0dc8meaabaqaciaacaGaaeqabaqabeGadaaakeaadaWcaaqaaiabgkHiTiabdggaHnaaBaaaleaacqWGHbqyaeqaaOGaem4yamMaemiDaqNaemyDauhabaGaeGOmaiJafm4DaCNbaebadaWgaaWcbaGaemOrayKaem4uamfabeaaaaGcdaqadaqaaiabigdaXiabgkHiTiabdQha6naabmaabaGaeGymaeJaeyOeI0YaaeWaaeaacuWGHbqygaqeaiabgUcaRiabdggaHnaaBaaaleaacqaI0aancqWGgbGrcqWGtbWuaeqaaaGccaGLOaGaayzkaaGaei4la8IaeGOmaidacaGLOaGaayzkaaaacaGLOaGaayzkaaaaaa@4C4D@
Δu between groups (Δu_b_)	aatu4w¯HS(−c+z(1+c(1−(a¯+a2HS)))) MathType@MTEF@5@5@+=feaafiart1ev1aaatCvAUfKttLearuWrP9MDH5MBPbIqV92AaeXatLxBI9gBaebbnrfifHhDYfgasaacH8akY=wiFfYdH8Gipec8Eeeu0xXdbba9frFj0=OqFfea0dXdd9vqai=hGuQ8kuc9pgc9s8qqaq=dirpe0xb9q8qiLsFr0=vr0=vr0dc8meaabaqaciaacaGaaeqabaqabeGadaaakeaadaWcaaqaaiabdggaHnaaBaaaleaacqWGHbqyaeqaaOGaemiDaqNaemyDauhabaGaeGinaqJafm4DaCNbaebadaWgaaWcbaGaemisaGKaem4uamfabeaaaaGcdaqadaqaaiabgkHiTiabdogaJjabgUcaRiabdQha6naabmaabaGaeGymaeJaey4kaSIaem4yam2aaeWaaeaacqaIXaqmcqGHsisldaqadaqaaiqbdggaHzaaraGaey4kaSIaemyyae2aaSbaaSqaaiabikdaYiabdIeaijabdofatbqabaaakiaawIcacaGLPaaaaiaawIcacaGLPaaaaiaawIcacaGLPaaaaiaawIcacaGLPaaaaaa@4E2C@	aatu2w¯FS(−c+z(1+c(1−(a¯+a2HS)))) MathType@MTEF@5@5@+=feaafiart1ev1aaatCvAUfKttLearuWrP9MDH5MBPbIqV92AaeXatLxBI9gBaebbnrfifHhDYfgasaacH8akY=wiFfYdH8Gipec8Eeeu0xXdbba9frFj0=OqFfea0dXdd9vqai=hGuQ8kuc9pgc9s8qqaq=dirpe0xb9q8qiLsFr0=vr0=vr0dc8meaabaqaciaacaGaaeqabaqabeGadaaakeaadaWcaaqaaiabdggaHjabdggaHjabdsha0jabdwha1bqaaiabikdaYiqbdEha3zaaraWaaSbaaSqaaiabdAeagjabdofatbqabaaaaOWaaeWaaeaacqGHsislcqWGJbWycqGHRaWkcqWG6bGEdaqadaqaaiabigdaXiabgUcaRiabdogaJnaabmaabaGaeGymaeJaeyOeI0YaaeWaaeaacuWGHbqygaqeaiabgUcaRiabdggaHnaaBaaaleaacqaIYaGmcqWGibascqWGtbWuaeqaaaGccaGLOaGaayzkaaaacaGLOaGaayzkaaaacaGLOaGaayzkaaaacaGLOaGaayzkaaaaaa@4DEE@
Δu total (Δu_t_)	aatu(−c+z(14+c(1−(3a¯4+a2HS2)))) MathType@MTEF@5@5@+=feaafiart1ev1aaatCvAUfKttLearuWrP9MDH5MBPbIqV92AaeXatLxBI9gBaebbnrfifHhDYfgasaacH8akY=wiFfYdH8Gipec8Eeeu0xXdbba9frFj0=OqFfea0dXdd9vqai=hGuQ8kuc9pgc9s8qqaq=dirpe0xb9q8qiLsFr0=vr0=vr0dc8meaabaqaciaacaGaaeqabaqabeGadaaakeaacqWGHbqydaWgaaWcbaGaemyyaegabeaakiabdsha0jabdwha1naabmaabaGaeyOeI0Iaem4yamMaey4kaSIaemOEaO3aaeWaaeaadaWcaaqaaiabigdaXaqaaiabisda0aaacqGHRaWkcqWGJbWydaqadaqaaiabigdaXiabgkHiTmaabmaabaWaaSaaaeaacqaIZaWmcuWGHbqygaqeaaqaaiabisda0aaacqGHRaWkdaWcaaqaaiabdggaHnaaBaaaleaacqaIYaGmcqWGibascqWGtbWuaeqaaaGcbaGaeGOmaidaaaGaayjkaiaawMcaaaGaayjkaiaawMcaaaGaayjkaiaawMcaaaGaayjkaiaawMcaaaaa@4D1B@	aatu(−c+z(12+c(1−(a¯2+a2HS)))) MathType@MTEF@5@5@+=feaafiart1ev1aaatCvAUfKttLearuWrP9MDH5MBPbIqV92AaeXatLxBI9gBaebbnrfifHhDYfgasaacH8akY=wiFfYdH8Gipec8Eeeu0xXdbba9frFj0=OqFfea0dXdd9vqai=hGuQ8kuc9pgc9s8qqaq=dirpe0xb9q8qiLsFr0=vr0=vr0dc8meaabaqaciaacaGaaeqabaqabeGadaaakeaacqWGHbqydaWgaaWcbaGaemyyaegabeaakiabdsha0jabdwha1naabmaabaGaeyOeI0Iaem4yamMaey4kaSIaemOEaO3aaeWaaeaadaWcaaqaaiabigdaXaqaaiabikdaYaaacqGHRaWkcqWGJbWydaqadaqaaiabigdaXiabgkHiTmaabmaabaWaaSaaaeaacuWGHbqygaqeaaqaaiabikdaYaaacqGHRaWkcqWGHbqydaWgaaWcbaGaeGOmaiJaemisaGKaem4uamfabeaaaOGaayjkaiaawMcaaaGaayjkaiaawMcaaaGaayjkaiaawMcaaaGaayjkaiaawMcaaaaa@4B1D@

Clearly, policing decreases the benefit of competition to within-group fitness, and decreases the deleterious effect of competition on between-group fitness; however, this group-level benefit is mediated by the cost of policing. The direction and magnitude of among-group selection on policing effort (Table 9) reflects these opposing pressures. For both half and full sib structured populations, increased policing is favored by selection among groups when the baseline level of policing [a_0_] is small – that is, when a0<(z−c2cz+1−aa(12+3u)2)
 MathType@MTEF@5@5@+=feaafiart1ev1aaatCvAUfKttLearuWrP9MDH5MBPbIqV92AaeXatLxBI9gBaebbnrfifHhDYfgasaacH8akY=wiFfYdH8Gipec8Eeeu0xXdbba9frFj0=OqFfea0dXdd9vqai=hGuQ8kuc9pgc9s8qqaq=dirpe0xb9q8qiLsFr0=vr0=vr0dc8meaabaqaciaacaGaaeqabaqabeGadaaakeaacqWGHbqydaWgaaWcbaGaeGimaadabeaakiabgYda8maabmaabaWaaSaaaeaacqWG6bGEcqGHsislcqWGJbWyaeaacqaIYaGmcqWGJbWycqWG6bGEaaGaey4kaSYaaSaaaeaacqaIXaqmcqGHsislcqWGHbqydaWgaaWcbaGaemyyaegabeaakmaabmaabaWaaSGaaeaacqaIXaqmaeaacqaIYaGmaaGaey4kaSIaeG4mamJaemyDauhacaGLOaGaayzkaaaabaGaeGOmaidaaaGaayjkaiaawMcaaaaa@4682@, and all other values are between 0 and 1.

Restricting this analysis to small values of a_a_, this result reduces to:

a0<zc−c+z2cz
 MathType@MTEF@5@5@+=feaafiart1ev1aaatCvAUfKttLearuWrP9MDH5MBPbIqV92AaeXatLxBI9gBaebbnrfifHhDYfgasaacH8akY=wiFfYdH8Gipec8Eeeu0xXdbba9frFj0=OqFfea0dXdd9vqai=hGuQ8kuc9pgc9s8qqaq=dirpe0xb9q8qiLsFr0=vr0=vr0dc8meaabaqaciaacaGaaeqabaqabeGadaaakeaacqWGHbqydaWgaaWcbaGaeGimaadabeaakiabgYda8maalaaabaGaemOEaONaem4yamMaeyOeI0Iaem4yamMaey4kaSIaemOEaOhabaGaeGOmaiJaem4yamMaemOEaOhaaaaa@3B54@

Increasing the cost of policing [c] decreases the value of the right hand side (RHS) of the eq. [10], limiting the parameter space for which increased policing is favored by between-group selection. In contrast, increasing the fixed level of competition, [z] increases the RHS of eq. [10], increasing the parameter space under which increased policing is favored by between-group selection. *Thus, increased competition within groups facilitates the evolution of policing by between-group selection*.

A comparison of Δ*u*_*amongHS *_with Δ*u*_*amongFS *_shows that among-group selection favoring increased policing effort is approximately twice as strong for full sib families as it is for half sib families (Table 9, row 4). The exact difference between Δ*u*_*amongFS *_with Δ*u*_*amongHS *_is ΔuamongHS2(1+czσa_AF_HS2w¯HS)
 MathType@MTEF@5@5@+=feaafiart1ev1aaatCvAUfKttLearuWrP9MDH5MBPbIqV92AaeXatLxBI9gBaebbnrfifHhDYfgasaacH8akY=wiFfYdH8Gipec8Eeeu0xXdbba9frFj0=OqFfea0dXdd9vqai=hGuQ8kuc9pgc9s8qqaq=dirpe0xb9q8qiLsFr0=vr0=vr0dc8meaabaqaciaacaGaaeqabaqabeGadaaakeaadaWcaaqaaiabfs5aejabdwha1naaBaaaleaacqWGHbqycqWGTbqBcqWGVbWBcqWGUbGBcqWGNbWzcqWGibascqWGtbWuaeqaaaGcbaGaeGOmaidaaiabcIcaOiabigdaXiabgUcaRmaalaaabaGaem4yamMaemOEaOhcciGae83Wdm3aa0baaSqaaiabdggaHjabc+faFjabdgeabjabdAeagjabc+faFjabdIeaijabdofatbqaaiabikdaYaaaaOqaaiqbdEha3zaaraWaaSbaaSqaaiabdIeaijabdofatbqabaaaaOGaeiykaKcaaa@4F75@. The most important qualitative result from our derivation is that policing can be favored by between group selection and increasing relatedness (while keeping all other variables constant) strengthens the force of among-group selection favoring policing.

While among-group selection can favor increased policing, within group selection always favors reduced levels of policing (Table 9) Note that the claim that costless policing does not involve kin selection [30] is not substantiated by our model: when the cost of policing (c) is set to 0, there is no selection within groups and policing evolves solely by among-group selection. The difference between Δu_withinFS _and Δu_withinHS _equals

1w¯FS(aactu4(1−z(1−a¯+aa(12−u))+ΔuamongHSczσa_AF_HS2w¯HS)
 MathType@MTEF@5@5@+=feaafiart1ev1aaatCvAUfKttLearuWrP9MDH5MBPbIqV92AaeXatLxBI9gBaebbnrfifHhDYfgasaacH8akY=wiFfYdH8Gipec8Eeeu0xXdbba9frFj0=OqFfea0dXdd9vqai=hGuQ8kuc9pgc9s8qqaq=dirpe0xb9q8qiLsFr0=vr0=vr0dc8meaabaqaciaacaGaaeqabaqabeGadaaakeaadaWcaaqaaiabigdaXaqaaiqbdEha3zaaraWaaSbaaSqaaiabdAeagjabdofatbqabaaaaOWaaeWaaeaadaWcaaqaaiabdggaHnaaBaaaleaacqWGHbqyaeqaaOGaem4yamMaemiDaqNaemyDauhabaGaeGinaqdaamaabmaabaGaeGymaeJaeyOeI0IaemOEaONaeiikaGIaeGymaeJaeyOeI0IafmyyaeMbaebacqGHRaWkcqWGHbqydaWgaaWcbaGaemyyaegabeaakmaabmaabaWaaSaaaeaacqaIXaqmaeaacqaIYaGmaaGaeyOeI0IaemyDauhacaGLOaGaayzkaaaacaGLOaGaayzkaaGaey4kaSYaaSaaaeaacqqHuoarcqWG1bqDdaWgaaWcbaGaemyyaeMaemyBa0Maem4Ba8MaemOBa4Maem4zaCMaemisaGKaem4uamfabeaakiabdogaJjabdQha6HGaciab=n8aZnaaDaaaleaacqWGHbqycqGGFbWxcqWGbbqqcqWGgbGrcqGGFbWxcqWGibascqWGtbWuaeaacqaIYaGmaaaakeaacuWG3bWDgaqeamaaBaaaleaacqWGibascqWGtbWuaeqaaaaaaOGaayjkaiaawMcaaaaa@6CEC@

It can be shown that Δu_withinFS _is greater than Δu_withinHS _when z<11−a¯
 MathType@MTEF@5@5@+=feaafiart1ev1aaatCvAUfKttLearuWrP9MDH5MBPbIqV92AaeXatLxBI9gBaebbnrfifHhDYfgasaacH8akY=wiFfYdH8Gipec8Eeeu0xXdbba9frFj0=OqFfea0dXdd9vqai=hGuQ8kuc9pgc9s8qqaq=dirpe0xb9q8qiLsFr0=vr0=vr0dc8meaabaqaciaacaGaaeqabaqabeGadaaakeaacqWG6bGEcqGH8aapdaWcaaqaaiabigdaXaqaaiabigdaXiabgkHiTiqbdggaHzaaraaaaaaa@336D@. Since within-group selection decreases the frequency of a policing allele, and since z cannot be greater than one unless there is already a significant level of policing in the population, selection against policing is initially stronger in populations consisting of half-sib families than it is in populations of full-sib families, but as the level of policing increases this difference diminishes. This result partially explains why half-sib families can sustain high levels of policing – within-group selection against high levels of policing is weaker in half-sib based populations than in full-sib based populations.

**Figure 3:**
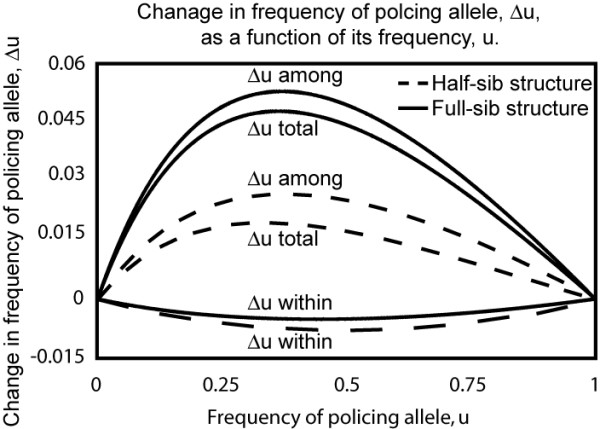
**Change in frequency (Δu) of a 'policing' policing allele due to selection within and among groups**. The sum of selection within and among groups equals the total change in allele frequency (Δu total). The policing allele has an additive effect (a_a_) of 0.2, and a cost (c) of 0.2. The population begins with no baseline level of policing (a_0 _= 0) and a 0.8 level of competition (z). q* as a function of the base-line level of competition (z_0_) and the additive

The difference between populations structured as full and half-sib groups in the total change in policing-allele frequency is the sum the differences between them from selection within and between groups. Under most parameter values, policing is more strongly favored in full-sib than in half-sib structured populations (Figure 3); again, this is contrary to the beliefs that reduced relatedness favors the evolution of policing and that the evolution of policing is a process distinct from kin selection.

#### Solving for optima

Frank [13] found that the co-evolutionary system of competition and policing moves towards one of two equilibria, depending on the cost of policing and the population genetic structure. When relatedness is greater than one minus the cost of policing, Frank's model predicts that there will be no policing and the level of competition (z*) will equal one minus relatedness. From our derivation of Δu_total_, we find that an allele that increases policing effort will only increase in frequency when c<z2−2z+3za0z+zaa+4uzaa
 MathType@MTEF@5@5@+=feaafiart1ev1aaatCvAUfKttLearuWrP9MDH5MBPbIqV92AaeXatLxBI9gBaebbnrfifHhDYfgasaacH8akY=wiFfYdH8Gipec8Eeeu0xXdbba9frFj0=OqFfea0dXdd9vqai=hGuQ8kuc9pgc9s8qqaq=dirpe0xb9q8qiLsFr0=vr0=vr0dc8meaabaqaciaacaGaaeqabaqabeGadaaakeaacqWGJbWycqGH8aapdaWcaaqaaiabdQha6bqaaiabikdaYiabgkHiTiabikdaYiabdQha6jabgUcaRiabiodaZiabdQha6jabdggaHnaaBaaaleaacqaIWaamaeqaaOGaemOEaONaey4kaSIaemOEaONaemyyae2aaSbaaSqaaiabdggaHbqabaGccqGHRaWkcqaI0aancqWG1bqDcqWG6bGEcqWGHbqydaWgaaWcbaGaemyyaegabeaaaaaaaa@48CE@ for full sib families, and when c<z4−4z+5za0+zaa+8uzaa
 MathType@MTEF@5@5@+=feaafiart1ev1aaatCvAUfKttLearuWrP9MDH5MBPbIqV92AaeXatLxBI9gBaebbnrfifHhDYfgasaacH8akY=wiFfYdH8Gipec8Eeeu0xXdbba9frFj0=OqFfea0dXdd9vqai=hGuQ8kuc9pgc9s8qqaq=dirpe0xb9q8qiLsFr0=vr0=vr0dc8meaabaqaciaacaGaaeqabaqabeGadaaakeaacqWGJbWycqGH8aapdaWcaaqaaiabdQha6bqaaiabisda0iabgkHiTiabisda0iabdQha6jabgUcaRiabiwda1iabdQha6jabdggaHnaaBaaaleaacqaIWaamaeqaaOGaey4kaSIaemOEaONaemyyae2aaSbaaSqaaiabdggaHbqabaGccqGHRaWkcqaI4aaocqWG1bqDcqWG6bGEcqWGHbqydaWgaaWcbaGaemyyaegabeaaaaaaaa@4765@ for half sib families.

Inserting values of z* found in the previous section (1/2 for full sib and 3/4 for half sib families) for z, an allele which increases policing effort can increase in frequency when c<12+3a0+aa+4uaa
 MathType@MTEF@5@5@+=feaafiart1ev1aaatCvAUfKttLearuWrP9MDH5MBPbIqV92AaeXatLxBI9gBaebbnrfifHhDYfgasaacH8akY=wiFfYdH8Gipec8Eeeu0xXdbba9frFj0=OqFfea0dXdd9vqai=hGuQ8kuc9pgc9s8qqaq=dirpe0xb9q8qiLsFr0=vr0=vr0dc8meaabaqaciaacaGaaeqabaqabeGadaaakeaacqWGJbWycqGH8aapdaWcaaqaaiabigdaXaqaaiabikdaYiabgUcaRiabiodaZiabdggaHnaaBaaaleaacqaIWaamaeqaaOGaey4kaSIaemyyae2aaSbaaSqaaiabdggaHbqabaGccqGHRaWkcqaI0aancqWG1bqDcqWGHbqydaWgaaWcbaGaemyyaegabeaaaaaaaa@3EF1@ or c<34+15a0+3aa+24uaa
 MathType@MTEF@5@5@+=feaafiart1ev1aaatCvAUfKttLearuWrP9MDH5MBPbIqV92AaeXatLxBI9gBaebbnrfifHhDYfgasaacH8akY=wiFfYdH8Gipec8Eeeu0xXdbba9frFj0=OqFfea0dXdd9vqai=hGuQ8kuc9pgc9s8qqaq=dirpe0xb9q8qiLsFr0=vr0=vr0dc8meaabaqaciaacaGaaeqabaqabeGadaaakeaacqWGJbWycqGH8aapdaWcaaqaaiabiodaZaqaaiabisda0iabgUcaRiabigdaXiabiwda1iabdggaHnaaBaaaleaacqaIWaamaeqaaOGaey4kaSIaeG4mamJaemyyae2aaSbaaSqaaiabdggaHbqabaGccqGHRaWkcqaIYaGmcqaI0aancqWG1bqDcqWGHbqydaWgaaWcbaGaemyyaegabeaaaaaaaa@41D3@ for full and half sib based populations, respectively. Beginning with no policing (a_0 _= 0), a rare mutant (u is small) with a small policing effort (a_a _is small and positive) will only increase when c < 1/2 for full sib, or c < 3/4 for half sib structured populations. This result is consistent with Frank's first equilibrium prediction – when r > 1 - c, policing will not invade and the system will be stable with z_ij _= z_i. _= z_.. _= z* = 1 - r, and a_ij _= a_i. _= a_.. _= a* = 0.

We can also solve for the equilibrium value of a_0_* with a fixed value of competition. For full sib structured populations, a0∗=23cz(cz−c+z2)
 MathType@MTEF@5@5@+=feaafiart1ev1aaatCvAUfKttLearuWrP9MDH5MBPbIqV92AaeXatLxBI9gBaebbnrfifHhDYfgasaacH8akY=wiFfYdH8Gipec8Eeeu0xXdbba9frFj0=OqFfea0dXdd9vqai=hGuQ8kuc9pgc9s8qqaq=dirpe0xb9q8qiLsFr0=vr0=vr0dc8meaabaqaciaacaGaaeqabaqabeGadaaakeaacqWGHbqydaqhaaWcbaGaeGimaadabaGaey4fIOcaaOGaeyypa0ZaaSaaaeaacqaIYaGmaeaacqaIZaWmcqWGJbWycqWG6bGEaaWaaeWaaeaacqWGJbWycqWG6bGEcqGHsislcqWGJbWycqGHRaWkdaWcaaqaaiabdQha6bqaaiabikdaYaaaaiaawIcacaGLPaaaaaa@3FC5@ and for half sib structured populations a0∗=45cz(cz−c+z4)
 MathType@MTEF@5@5@+=feaafiart1ev1aaatCvAUfKttLearuWrP9MDH5MBPbIqV92AaeXatLxBI9gBaebbnrfifHhDYfgasaacH8akY=wiFfYdH8Gipec8Eeeu0xXdbba9frFj0=OqFfea0dXdd9vqai=hGuQ8kuc9pgc9s8qqaq=dirpe0xb9q8qiLsFr0=vr0=vr0dc8meaabaqaciaacaGaaeqabaqabeGadaaakeaacqWGHbqydaqhaaWcbaGaeGimaadabaGaey4fIOcaaOGaeyypa0ZaaSaaaeaacqaI0aanaeaacqaI1aqncqWGJbWycqWG6bGEaaWaaeWaaeaacqWGJbWycqWG6bGEcqGHsislcqWGJbWycqGHRaWkdaWcaaqaaiabdQha6bqaaiabisda0aaaaiaawIcacaGLPaaaaaa@3FD1@. Subtracting a_0_* _HS _from a_0_* _FS _yields 215cz(c+z−cz)
 MathType@MTEF@5@5@+=feaafiart1ev1aaatCvAUfKttLearuWrP9MDH5MBPbIqV92AaeXatLxBI9gBaebbnrfifHhDYfgasaacH8akY=wiFfYdH8Gipec8Eeeu0xXdbba9frFj0=OqFfea0dXdd9vqai=hGuQ8kuc9pgc9s8qqaq=dirpe0xb9q8qiLsFr0=vr0=vr0dc8meaabaqaciaacaGaaeqabaqabeGadaaakeaadaWcaaqaaiabikdaYaqaaiabigdaXiabiwda1iabdogaJjabdQha6baadaqadaqaaiabdogaJjabgUcaRiabdQha6jabgkHiTiabdogaJjabdQha6bGaayjkaiaawMcaaaaa@3B52@, a number which is generally positive, providing further support for the claim that, all else equal, high relatedness favors higher levels of policing.

### Summary of Model 2: The Evolution of Policing Effort

We apply a family selection approach to the problem of the evolution of policing effort. Selection among families generally increases the frequency of an allele that increases policing effort, while selection within families always decreases the frequency of a policing allele. Under identical parameters, selection among half sib groups favoring a policing allele is twice as strong as the same selection among a population of half-sib groups. By inserting equilibrium values of z* from section 1A into our Δu expressions, we show that a nonzero level of policing can evolve only when c < 1/2 and 3/4 for full and half- sib families, respectively.

### Model 3: The Co-evolution of Policing and Competition

#### Population-genetic dynamics

In this model we examine the co-evolution of competition and policing behavior. In this two-locus model we retain the naming conventions in models 1 and 2. The fitness of the j^th ^in the i^th ^group is given in eq. [1]. The mean fitness of the i^th ^family

*w*_*i*. _= (1 - *ca*_*i*._)(1 - *z*_*i*_(1 - *a*_*i*._))

equals and the mean population fitness equals

w¯=(1−cai.)(1−zi(1−ai.))−σa_AF2
 MathType@MTEF@5@5@+=feaafiart1ev1aaatCvAUfKttLearuWrP9MDH5MBPbIqV92AaeXatLxBI9gBaebbnrfifHhDYfgasaacH8akY=wiFfYdH8Gipec8Eeeu0xXdbba9frFj0=OqFfea0dXdd9vqai=hGuQ8kuc9pgc9s8qqaq=dirpe0xb9q8qiLsFr0=vr0=vr0dc8meaabaqaciaacaGaaeqabaqabeGadaaakeaacuWG3bWDgaqeaiabg2da9maabmaabaGaeGymaeJaeyOeI0Iaem4yamMaemyyae2aaSbaaSqaaiabdMgaPjabc6caUaqabaaakiaawIcacaGLPaaadaqadaqaaiabigdaXiabgkHiTiabdQha6naaBaaaleaacqWGPbqAaeqaaOWaaeWaaeaacqaIXaqmcqGHsislcqWGHbqydaWgaaWcbaGaemyAaKMaeiOla4cabeaaaOGaayjkaiaawMcaaaGaayjkaiaawMcaaiabgkHiTGGaciab=n8aZnaaDaaaleaacqWGHbqycqGGFbWxcqWGbbqqcqWGgbGraeaacqaIYaGmaaaaaa@4DD7@

Family tables are presented in Additional file 2.

The results of this model parallel results from models 1B and 2. Mean population fitness, Δu within families, among families and total are equivalent to values in Table 9 with z¯
 MathType@MTEF@5@5@+=feaafiart1ev1aaatCvAUfKttLearuWrP9MDH5MBPbIqV92AaeXatLxBI9gBaebbnrfifHhDYfgasaacH8akY=wiFfYdH8Gipec8Eeeu0xXdbba9frFj0=OqFfea0dXdd9vqai=hGuQ8kuc9pgc9s8qqaq=dirpe0xb9q8qiLsFr0=vr0=vr0dc8meaabaqaciaacaGaaeqabaqabeGadaaakeaacuWG6bGEgaqeaaaa@2E41@ replacing z. Similarly, Δq within and among families as well as Δq total are equivalent to values listed in 6, with a¯
 MathType@MTEF@5@5@+=feaafiart1ev1aaatCvAUfKttLearuWrP9MDH5MBPbIqV92AaeXatLxBI9gBaebbnrfifHhDYfgasaacH8akY=wiFfYdH8Gipec8Eeeu0xXdbba9frFj0=OqFfea0dXdd9vqai=hGuQ8kuc9pgc9s8qqaq=dirpe0xb9q8qiLsFr0=vr0=vr0dc8meaabaqaciaacaGaaeqabaqabeGadaaakeaacuWGHbqygaqeaaaa@2E0F@ replacing a. No linkage disequilibrium is generated by selection either within or among groups, therefore the dynamics of the co-evolution of policing and competition do not depend on whether they are jointly polymorphic, or if the invasion of a new type occurs while the other locus is monomorphic. As in models 1 and 2, genetic constraints such as mutational step size, and dominance can prevent populations from attaining these equilibrium values.

#### Solving for optima

As in model 2, and as Frank [13,14] found, policing will only evolve when r < 1 - c. When policing is favored by selection, policing effort will rise above one, the biological cap on the level of policing. By constraining the system to avoid negative individual fitnesses (by keeping a < 1), when policing is favored it will approach one and the level of competition will approach that found in model 1B (above).

#### Population-genetic constraints

Notably, although this two-gene, two-trait model makes equivalent 'long-term' predictions as the single-trait based approaches, this model places additional constraints on the order and size of effect of mutations to competition and policing, which may prohibit a population from achieving the ultimate equilibria. That is large mutational many mutations at one locus, that place a population closer to long-term equilibria may be lost because the appropriate trait values at the other locus do not yet exist.

## Discussion and Conclusion

Conflict between the levels of selection is a classical problem of evolution theory [6,8,25,31-34]. Here, in formal evolutionary genetic models, we investigated the evolution of two traits, interference competition and policing, that, not only, interact to affect fitness but also are affected by selection within and between groups, albeit in opposite ways. The first trait, interference competition, increases the fitness of individuals within groups but does so at a cost to group fitness. The second trait, policing, increases group mean fitness but at a cost to the fitness of policing individuals within groups. By partitioning total selection into within and among group components, we showed how each level of selection contributes to the evolutionary dynamics and long-term evolutionary outcome of selection on policing and competition. We noted that costless policing involves only selection among groups.

**Figure 4:**
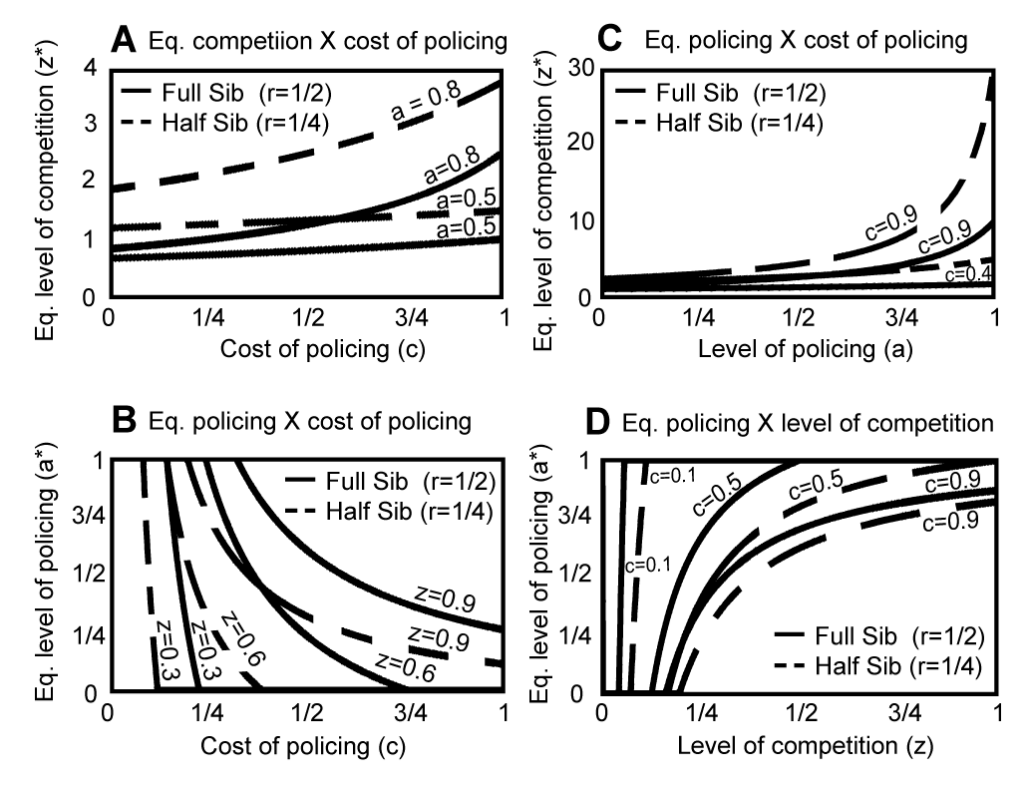
**Equilibrium values of competition and policing**. Equilibrium values of competition as a function of the level of policing (a) and the cost of policing (a) (figures 4A and 4C), and equilibrium values of policing as a function of the level of competition (z) and the cost of policing (c) (figures 4B and 4D).

We found that competition is opposed by among-group selection and decreases with higher levels of genetic relatedness: over all values of cost and for any given level of policing, there is a negative relationship between competition and relatedness. That is, under identical parameters, the equilibrium values of competition are always higher for half-sib than for full-sib structured populations (Fig. 4a). As has been shown before [13,14,27], kin selection opposes competition within kin groups. We also find that interference competition with policing can evolve to a level that exceeds its maximum value (z* = 1) without policing, a finding similar to that of Frank [13,14]: a high level of policing allows a high level of interference competition.

One of our novel findings is that policing is favoured by among-group selection and it reaches higher values with higher genetic relatedness within groups as we illustrate graphically (Fig. 4). Thus, kin selection favors the evolution of policing: across a range of costs of policing and for a given level of competition, the equilibrium level of policing is higher for full-sib than for half-sib structured populations (Fig. 4b). As well, the rate of increase of a policing allele increases with genetic relatedness (Fig. 3). We conclude that, although policing may be a different mechanism than altruism for ensuring group cooperation, it evolves by the same evolutionary process as altruism, namely, kin selection.

We also find that, owing to the multiplicative nature of the group-mean fitness function, the mean level of competition has a strong influence on the equilibrium level of policing and vice versa (Figs. 4c,d). However, the influences are not strictly reciprocal. The equilibrium level of policing cannot rise above one (where it renders all competition ineffective) and remains at zero until high values of competition exist, while the equilibrium level of competition can get very large and grow very quickly, especially when policing is costly. Together, these trait associations result in a negative relationship between the equilibrium values of policing and relatedness – the high levels of competition favored in populations with low relatedness create a strong selective pressure favoring policing behavior and overcome the weakness of kin selection in less genetically structured groups. Thus, *the joint equilibrium relationship between relatedness and policing is the opposite of the dynamical one*. It is this difference between the dynamic and equilibrium relationship to relatedness that has led some to interpret policing as an alternative to kin selection. Our dynamic model reveals the source of this confusion.

Our evolutionary genetic approach also reveals the existence of planes of stable, interior equilibria. For a fixed level of policing, there exists a plane of stable equilibria with competitive specialists and a less competitive, self-policing class (Figures 3 and 4). Similarly, the evolution of policing with a fixed level of competition gives rise to planes of stable gene frequency equilibria with policing and non-policing individuals (Brandvain unpublished). These non-policing individuals are cheaters or free-riders by virtue of the fact that they bear none of the costs of policing but reap the reward of reduced within-group resource competition. Together, these findings indicate that neither trait may reach the ultimate evolutionary outcome characterized by optimizing the mean fitness function. Our further analyses of the one-trait models, adding mutant alleles causing increased competition or reduced policing, respectively, result in the population moving from one region of stable equilibria to another, toward the ultimate outcomes predicted by Frank's model. Thus, our regions of stable equilibria are invasible by mutations and the ESS coincides with that predicted by Frank and his collaborators. However, rates of evolution can be very slow and depend upon the fortuitous input of a continuing series of mutations with effects of a particular kind occurring in the right order with respect to the equilibrium. Thus, when both traits are evolving simultaneously, there is reason to question whether the ESS can be achieved.

We used a two-locus model to investigate the simultaneous evolution of competition and policing. Like individual selection models with multiplicative fitnesses, selection in this model does not generate linkage disequilibrium between the competition and policing loci. As a result, the evolution of each trait is affected by the genotypic mean value of the other (playing the role of the fixed levels in the one locus model). Hence, we find similar regions of stable two-locus equilibria, with mixed levels of both competition and policing behaviors. Similarly, we find that mutant alleles at either locus can invade these stable equilibria. However, depending on the order and magnitude of the mutational effects introduced, the population can move either toward or away from the ultimate evolutionary outcome. Thus, achieving the joint ESS depends not only on the occurrence of mutations for policing and competitive ability but also on the mutations occurring in the right order, a much more onerous requirement than in the one-locus models. As a result, a population's sojourn away from the ESS may be extremely long and we should expect diversity among species (or among populations of the same species) in levels of competition and policing.

### Biological Implications

In some circumstances, different genes can experience different values of relatedness. For example, maternally inherited mitochondrial genes can be more closely related than diploid nuclear genes and the mating system can change both the absolute and relative values [35]. In genomic imprinting (the differential expression of a gene based on its parent of origin), a similar situation arises within the broods of multiply mated females: siblings are more closely related for maternally derived alleles than they are for paternally derived alleles. High relatedness of paternally derived alleles in monogamous or inbred sib-cohorts selects for self restraint of paternally derived competitive alleles, while low relatedness of siblings from multiple mated mothers will select for increased levels of competitive effort of paternally derived alleles [36]. Because our model allows for the separation of probability of ibd by parent of origin and because we show that policing is favored by selection between groups, we predict that the high competition of paternally-derived alleles will result in increased policing by maternally derived alleles.

Because genes in separate species are not closely related, policing has been invoked in this way: "the study of interspecific mutualistic associations offers the opportunity to explore the mechanisms ...that maintain cooperative behavior even in the absence of kin selection" ([37] p. 254; see also [13] p. 520). This view of policing has motivated the use of non-genetic, economic-optimum models of co-evolution to understand the evolution of symbioses. The evolution of conflict reduction without relatedness appears to provide an important alternative explanation for major transitions in evolution. In this view, policing (sometimes called sanctioning) evolves under very low relatedness and is an alternative to kin selection, which also favors decreased competitive ability. We have shown that policing is not an alternative to kin-group selection, thus, although policing could be an important force in the evolution of symbioses [38], especially in cases of co-inheritance [7,35,39], it does not offer a new class of evolutionary explanation.

While the population genetic models of policing and competition presented in this paper highlight both the dynamics of the evolution and the conflicting forces of selection within and between groups, Frank's models are more analytically tractable and both models make the same predictions about the long term evolutionary outcome of natural selection. Thus, these complementary approaches provided a clearer view of the evolutionary forces involved in competition and policing behaviors.

## Authors' contributions

YB and MJW conceived of the research and co-wrote the paper. YB conducted the theoretical research, and produced the figures and tables.

## Supplementary Material

Additional file 1 Exact solution for Δq, half sib families. Here we show the deviation between results assuming Hardy-Weinberg Equilibrium, and the exact solution without this assumption. Click here for file

Additional file 3 Invasibility of a 3^rd ^allele in a full-sib competition model. Here we present the invasion dynamics of a rare mutant entering a population with a stable two-locus polymorphism. Click here for file

Additional file 2 Two-locus family tales. Family tables for full and half sib families with both two alleles segregating at each locus. Click here for file
