# Supplementary material for: The evolution of competition and policing: opposing selection within and among groups
Source: BMC Evol Biol. 2007 Oct 25;7:203. doi: 10.1186/1471-2148-7-203 (PMC2222249; doi:10.1186/1471-2148-7-203)
Supplement: Additional file 1 — Exact solution for Δq, half sib families. Here we show the deviation between results assuming Hardy-Weinberg Equilibrium, and the exact solution without this assumption. [file 1471-2148-7-203-S1.doc]

Appendix1 The exact solution for the evolution of competitive behavior (Model 1A) in a population of half-sib families.

In the exact solution, we do not enforce Hardy-Weinberg genotypic frequencies to the frequencies of maternal families. Therefore, assuming no difference in allele frequencies between the sexes, A_0_ homozygous mothers occur in frequency $f_{A_{0}A_{0}}$, heterozygous mothers in frequency $f_{A_{0}A_{1}}$and A_1_ homozygous mothers in frequency $f_{A_{1}A_{1}}$. Remembering that , , and that , the genotypic frequencies in the next generation equal:

,

and Δq =

Notably, the approximate and exact models yield the same solution of z*, and thus the long-term dynamics of this system are unaffected by use of the approximate solution. Subtracting Δq_approximate_ from Δq_exact_ yields:

In figure A1 we plot Δq_exact_ and Δq_approximate_ as a function of time. With small values of za (such as 0.01 in the figA1) the difference between Δq_exact_ and Δq_approximate_ is very small.

Figure 1A. Δq approximate vs Δq exact, half-sib competition model.
