# Supplementary material for: The evolution of competition and policing: opposing selection within and among groups
Source: BMC Evol Biol. 2007 Oct 25;7:203. doi: 10.1186/1471-2148-7-203 (PMC2222249; doi:10.1186/1471-2148-7-203)
Supplement: Additional file 3 — Invasibility of a 3rd allele in a full-sib competition model. Here we present the invasion dynamics of a rare mutant entering a population with a stable two-locus polymorphism. [file 1471-2148-7-203-S3.doc]

Appendix 3: Invasibility of a 3^rd^ allele in a full-sib competition model

We examined the invasibility of a third allele in population at an intermediate, equilibrium frequency of p & q (q = q*) in a full sib competition model. The third allele, α, is found in frequency r. The additive effect of the α allele is z_a_ + δ. The phenotypes of individuals with at least on α allele are: z_Aα_= z_0_ + z_a_ + δ, z_aα_= z_0_ + 2z_a_ + δ, and z_aa_= z_0_ + 2(z_a_ + δ).

When the α allele is rare, and δ is small and positive, r increases in frequency when z_a_ is below the equilibrium value z* and decreases in frequency when it is above the z*. In contrast, when δ is small and negative, r decreases in frequency when z_a_ is below the equilibrium value z* and increases in frequency when it is above the z* (Appendix 3 Fig 1).

The family table including family type, family frequencies, offspring genotypes, family means and the change in frequency of r within a given family is given below (Appendix 3. Table 1).

Mean level of competition,

Population-mean fitness,

Selection among-families decreases the frequency of the third allele.

Selection within-families increases the frequency of the alternative, more competitive, third allele:

And the total change in the frequency of the third allele equals the sum of these values.


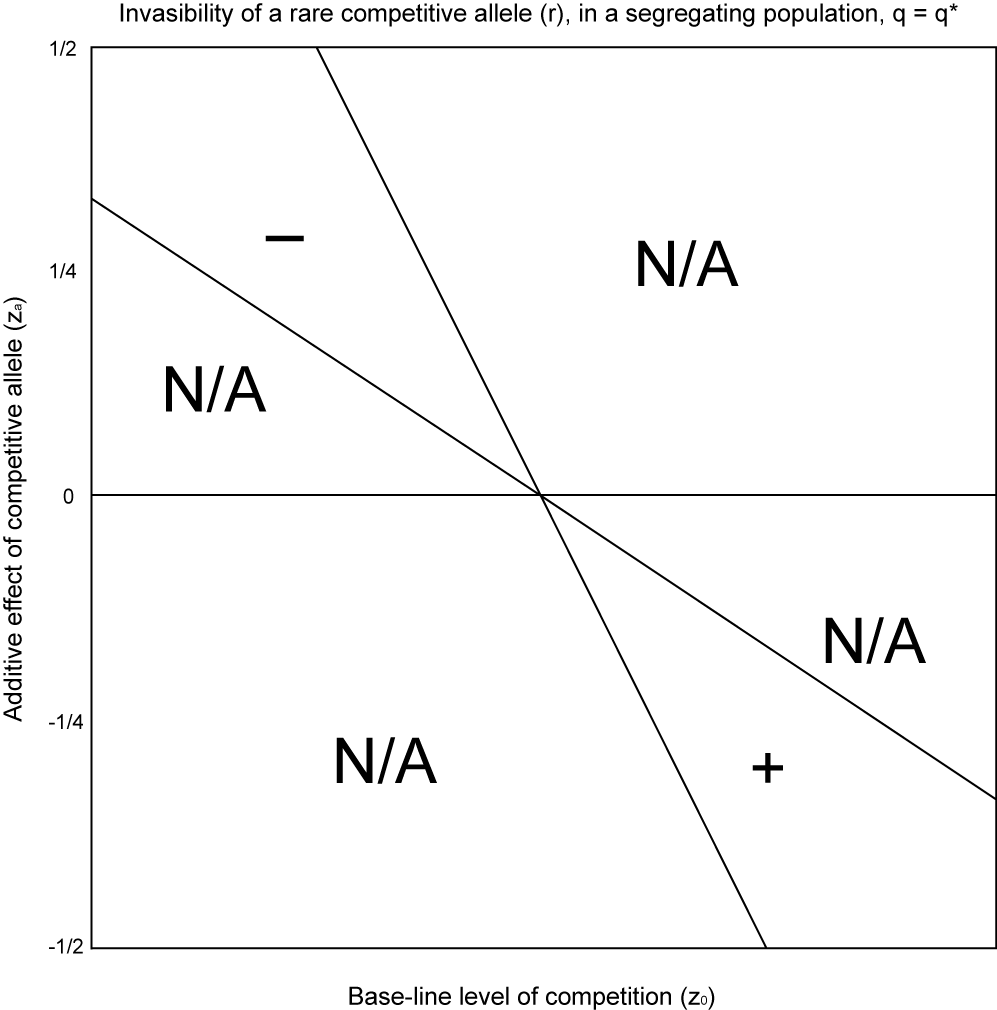


Appendix 3, Figure 1:

Invasibility of allele a in a full-sib structured population. Initially q = q*, and p = 1 – q*. The signs in the figure to the right show the direction of frequency change of allele when it is rare (r = 10^-15^) and δ is small and positive (δ = 0.001). Making δ small and negative changes the direction of allele frequency change.

Appendix 3. Table 1: Family frequency, fitness, and change in frequency of a second competitive allele a (which occurs in frequency r) for full sib _(fs)_ competition model, no policing.

|  | P1 | P2 | Fi | Kids | | | | | | Family means | | | |
| --- | --- | --- | --- | --- | --- | --- | --- | --- | --- | --- | --- | --- | --- |
|  |  |  |  | AA | Aa | Aα | αa | aa | aa | ri | zi | wi | Δri |
| 1 | AA | AA | p^4^ | 1 | 0 | 0 | 0 | 0 | 0 | 0 | z_0_ | 1-z_1FS_ | 0 |
| 2 | AA | Aa | 4p^3^q | 1/2 | 1/2 | 0 | 0 | 0 | 0 | 0 | z_0_+z_a_/2 | 1-_z2FS_ | 0 |
| 3 | AA | Aα | 4p^3^r | 1/2 | 0 | 1/2 | 0 | 0 | 0 | 1/4 | z_0_+(z_a_+δ)/2 | 1-_z3FS_ | (z_a_+δ)/(8z_3FS_) |
| 4 | AA | αa | 4p^2^rq | 0 | 1/2 | 1/2 | 0 | 0 | 0 | 1/4 | z_0_+z_a_+ δ/2 | 1-_z4FS_ | δ /(8z_4FS_) |
| 5 | AA | aa | 2p^2^r^2^ | 0 | 0 | 1 | 0 | 0 | 0 | 1/2 | z_0_+z_a_+ δ | 1-_z5FS_ | 0 |
| 6 | AA | aa | 2p^2^q^2^ | 0 | 1 | 0 | 0 | 0 | 0 | 0 | z_0_+z_a_ | 1-_z6FS_ | 0 |
| 7 | Aa | Aa | 4p^2^q^2^ | 1/4 | 1/2 | 0 | 0 | 0 | 1/4 | 0 | z_0_+z_a_ | 1-_z7FS_ | 0 |
| 8 | Aa | Aα | 8p^2^qr | 1/4 | 1/4 | 1/4 | 1/4 | 0 | 0 | 1/4 | z_0_+z_a_+δ/2 | 1-z_8FS_ | (z_a_+δ)/(8z_8FS_) |
| 9 | Aa | αa | 8q^2^pr | 0 | 1/4 | 1/4 | 1/4 | 0 | 1/4 | 1/4 | z_0_+(3z_a_+δ)/2 | 1-z_9FS_ | δ/(8z_9FS_) |
| 10 | Aa | aa | 4r^2^pq | 0 | 0 | 1/2 | 1/2 | 0 | 0 | 1/2 | z_0_+3z_a_/2+δ | 1-z_10FS_ | 0 |
| 11 | Aa | aa | 4q^3^p | 0 | 1/2 | 0 | 0 | 0 | 1/2 | 0 | z_0_+3z_a_/2 | 1-z_11FS_ | 0 |
| 12 | Aα | Aα | 4p^2^r^2^ | 1/4 | 0 | 1/2 | 0 | 1/4 | 0 | 1/2 | z_0_+z_a_+δ | 1-z_12FS_ | (z_a_+δ)/(4*z_12FS_) |
| 13 | Aα | αa | 8r^2^pq | 0 | 1/4 | 1/4 | 1/4 | 1/4 | 0 | 1/2 | z_0_+3z_a_/2+δ | 1-z_13FS_ | (z_a_+2δ)/(8*z_13FS_) |
| 14 | Aα | aa | 4r^3^p | 0 | 0 | 1/2 | 0 | 1/2 | 0 | 3/4 | z_0_+3(z_a_+δ)/2 | 1-z_14FS_ | (z_a_+δ)/(8z_14FS_) |
| 15 | Aα | aa | 4q^2^pr | 0 | 1/2 | 0 | 1/2 | 0 | 0 | 1/4 | z_0_+(3z_a_+δ)/2 | 1-z_15FS_ | (z_a_+δ)/(8z_15FS_) |
| 16 | αa | αa | 4q^2^r^2^ | 0 | 0 | 0 | 1/2 | 1/4 | 1/4 | 1/2 | z_0_+2z_a_+δ | 1-z_16FS_ | δ /(4z_16FS_) |
| 17 | αa | aa | 4qr^3^ | 0 | 0 | 0 | 1/2 | 1/2 | 0 | 3/4 | z_0_+2z_a_+3δ/2 | 1-z_17FS_ | δ/(8z_17FS_) |
| 18 | αa | aa | 4q^3^r | 0 | 0 | 0 | 1/2 | 0 | 1/2 | 1/4 | z_0_+2z_a_+δ/2 | 1-z_18FS_ | δ/(8z_18FS_) |
| 19 | aa | aa | r^4^ | 0 | 0 | 0 | 0 | 1 | 0 | 1 | z_0_+2(z_a_+δ) | 1-z_19FS_ | 0 |
| 20 | aa | aa | 2r^2^q^2^ | 0 | 0 | 0 | 1 | 0 | 0 | 1/2 | z_0_+2z_a_+δ | 1-z_20FS_ | 0 |
| 21 | aa | aa | q^4^ | 0 | 0 | 0 | 0 | 0 | 1 | 0 | z_0_+2z_a_ | 1-z_21FS_ | 0 |
